# Immunogenicity of dupilumab in adult and pediatric patients with atopic dermatitis

**DOI:** 10.3389/fimmu.2024.1466372

**Published:** 2024-11-11

**Authors:** Mohamed A. Kamal, Matthew P. Kosloski, Ching-Ha Lai, Michael A. Partridge, Manoj Rajadhyaksha, Vanaja Kanamaluru, Ashish Bansal, Arsalan Shabbir, Brad Shumel, Marius Ardeleanu, Susan M. Richards, Hong Yan, Christine R. Xu, Ainara Rodríguez-Marco, Jing Xiao, Faisal A. Khokhar, Guy Gherardi, Elisa Babilonia, Jennifer Maloney, Eric Mortensen, Bolanle Akinlade, Ned Braunstein, Neil Stahl, Albert Torri, John D. Davis, A. Thomas DiCioccio

**Affiliations:** ^1^ Regeneron Pharmaceuticals Inc., Tarrytown, NY, United States; ^2^ Sanofi, Bridgewater, NJ, United States; ^3^ Sanofi, Madrid, Spain; ^4^ Sanofi, Reading, United Kingdom

**Keywords:** anti-drug antibody, ADA, atopic dermatitis, dupilumab, immunogenicity, neutralizing antibody, NAb

## Abstract

**Background:**

Development of anti-drug antibodies (ADAs) and neutralizing antibodies (NAbs) to monoclonal antibodies may adversely impact pharmacokinetics, efficacy, and/or safety.

**Objective:**

To describe incidence, titer, and persistence of dupilumab ADAs and NAbs, and their effects on pharmacokinetics, efficacy, and safety in patients with atopic dermatitis (AD).

**Methods:**

This analysis included seven phase 3 randomized, placebo-controlled (N=2,992) and two long-term open-label extension (N=2,287) trials of subcutaneous dupilumab in adults and pediatric patients with moderate-to-severe AD. ADA, NAb, and dupilumab concentration in serum were assessed using validated immunoassays. ADA impacts on efficacy (EASI) and safety were assessed.

**Results:**

Treatment-emergent ADAs were observed in up to 8.6% (aged ≥18 years), 16.0% (12-17 years), 5.3% (6-11 years), and 2.0% (6 months to 5 years) dupilumab-treated patients. Among dupilumab-treated patients, ≤3.7% had persistent responses, <1% had high titers (≥10,000), and ≤5.1% were NAb-positive. NAbs were more common in patients with moderate- and high-titer ADA responses. High-titer ADAs, while infrequent, were the variable most associated with lower dupilumab concentrations in serum and loss of efficacy, independent of NAb status. Efficacy was generally similar in ADA-positive and -negative patients. For most patients with high- or moderate-titer ADAs, titers decreased and efficacy improved over time with continued dupilumab treatment. ADA-positive and -negative patients had similar incidences of treatment-emergent and serious treatment-emergent adverse events. One patient with high-titer ADAs developed serum sickness.

**Conclusion:**

In patients with AD, ADAs and NAbs had minimal impact on dupilumab concentration, efficacy, and safety, except for high-titer ADAs in a small number of patients.

**Clinical trial registration:**

ClinicalTrials.gov, identifiers (NCT02277743, NCT02277769, NCT02260986, NCT02395133, NCT01949311, NCT03054428, NCT03345914, NCT02612454, and NCT03346434).

## Graphical Abstract

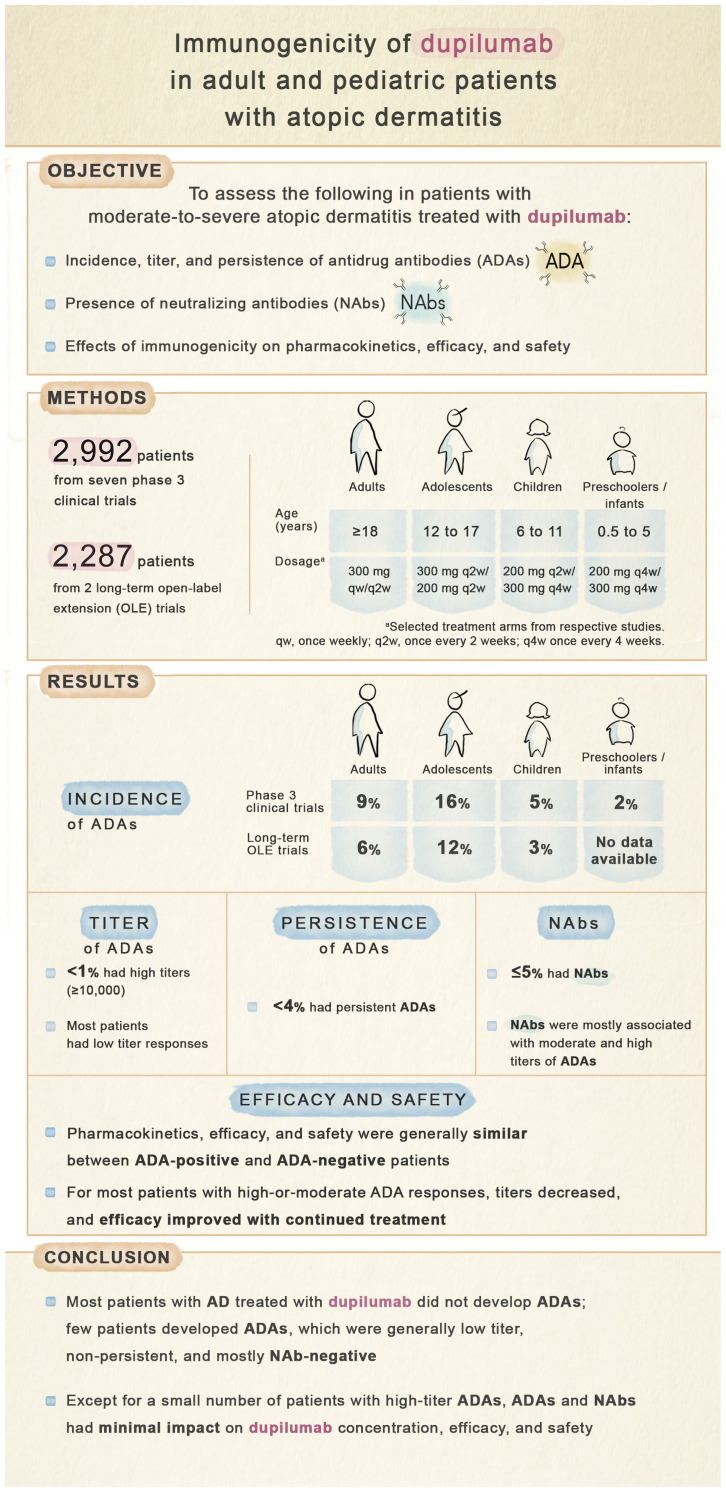

## 1 Introduction

Patients treated with therapeutic proteins may develop immunogenic responses and produce anti-drug antibodies (ADAs) (1). Neutralizing antibodies (NAbs), a subset of ADAs that bind to functional domains of proteins, may impair their biological activity and reduce efficacy. ADAs may also be associated with serious safety outcomes such as hypersensitivity reactions including serum sickness and anaphylaxis (2–8).

Dupilumab is a targeted, human *VelocImmune^®^-*derived monoclonal antibody (9, 10) that blocks interleukin-4 receptor alpha (IL-4Rα; shared receptor subunit for IL-4 and IL-13). Dupilumab inhibits signaling of both IL-4 and IL-13, which are key and central drivers of type 2 inflammation in multiple diseases (11, 12). Dupilumab is approved for type 2 inflammatory disorders including atopic dermatitis (AD), asthma, chronic rhinosinusitis with nasal polyps, eosinophilic esophagitis, and prurigo nodularis (13, 14).

This is the first comprehensive analysis of dupilumab immunogenicity in phase 3 clinical trials for patients 6 months of age to adults with AD. Although dupilumab is approved in multiple indications, this analysis of immunogenicity focuses on the AD indication, where the clinical trial population ranges from ages 6 months to adults, thus providing a robust analysis of immunogenicity spanning infants to adults. Herein we describe the incidence, titer, and persistence of ADA responses to dupilumab in the phase 3 clinical trials that supported indications in adult and pediatric populations with AD. Associations of immunogenic responses (i.e. ADAs and NAbs) with dupilumab pharmacokinetics (PK), efficacy, and safety are also assessed.

## 2 Methods

### 2.1 Study designs and treatment arms

This analysis included data from 9 randomized, placebo-controlled, double-blinded phase 3 clinical trials (RCTs) and open-label extensions (OLEs) of subcutaneous dupilumab in adults (aged ≥18 years), adolescents (12–17 years), children (6–11 years), and infants/preschoolers (6 months to 5 years) with moderate or severe AD (15–23). Study designs and baseline demographics and disease characteristics were previously described and are summarized in **Table 1** (15–23). Blood collection timepoints for assessment of ADAs and PK are provided in **Supplementary Table E1**.

**Table 1:** Clinical trials and treatment groups included in this analysis. (A) Adults. (B) Adolescents, children, and infants.

(A) Adults				
Trial	Patient population	Study design	Treatment period	Dose regimens (n) included in this analysis^a^
Pooled LIBERTY AD SOLO 1 (NCT02277743) and LIBERTY AD SOLO 2 (NCT02277769) (15)	Adults with moderate-to-severe AD	RCT	16 weeks	• Dupilumab 300 mg qw (n = 455) – Loading dose 600 mg • Dupilumab 300 mg q2w (n = 465) – Loading dose 600 mg • Placebo (n = 456)
LIBERTY AD CHRONOS (NCT02260986) (16)	Adults with moderate-to-severe AD	RCT	52 weeks	• Dupilumab 300 mg qw + TCS (n = 315) – Loading dose 600 mg • Dupilumab 300 mg q2w + TCS (n = 110) – Loading dose 600 mg • Placebo + TCS (n = 315)
LIBERTY AD SOLO-CONTINUE (NCT02395133) (19)^b^	Dupilumab-treated adults who had achieved IGA 0/1 or EASI-75 at week 16 in SOLO 1 or SOLO 2	RCT	36 weeks	• Dupilumab 300 mg qw (n = 87)^b^ • Dupilumab 300 mg q2w (n = 80)^b^ • Placebo (n = 82)
LIBERTY AD OLE (NCT01949311) (20)	Adult patients who had completed SOLO 1, SOLO 2, CHRONOS, or SOLO-CONTINUE	OLE	Up to 3 years; data cut-off for this analysis was December 1, 2018; median duration (Q1, Q3) of treatment in OLE, 93.0 weeks (68.7, 117.0)	• Dupilumab 300 mg qw; concomitant TCS or TCI permitted (n = 1,751) – Dupilumab was administered with a loading dose (600 mg) as the initial dupilumab dose in OLE for adult patients who had not received dupilumab in the previous 4 weeks

(B) Adolescents, children, and infants				
Trial	Patient population	Study design	Treatment period	Dose regimens (n) included in this analysis^a^
LIBERTY AD ADOL (NCT03054428) (17)	Adolescents (aged 12–17 years) with moderate-to-severe AD	RCT	16 weeks	• Dupilumab 200 mg q2w (baseline bodyweight <60 kg) (n = 43)^c^ – Loading dose 400 mg • Dupilumab 300 mg q2w (baseline bodyweight ≥60 kg) (n = 39)^b^ – Loading dose 600 mg • Placebo (n = 85)
LIBERTY AD PEDS (NCT03345914) (18)	Children (aged 6–11 years) with severe AD	RCT	16 weeks	• Dupilumab 200 mg q2w (baseline body weight ≥30 to <60 kg) + TCS (n = 59) – Loading dose 400 mg • Dupilumab 300 mg q4w (baseline body weight ≥15 to <60 kg) + TCS (n = 120) – Loading dose 600 mg • Placebo + TCS (n = 120)
LIBERTY AD PRESCHOOL (Part B: the phase 3 portion of a phase 2/3 trial; NCT03346434) (23)	Infants and preschool children (aged 6 months to 5 years) with moderate-to-severe AD	RCT	16 weeks	• Dupilumab 200 mg q4w + TCS (baseline body weight ≥5 to <15 kg) (n = 26)^d^ • Dupilumab 300 mg q4w + TCS (baseline body weight ≥15 to <30 kg) (n = 57)^d^ • Placebo + TCS (n = 78)
LIBERTY AD PED-OLE (NCT02612454) (21–23)	Adolescent patients who completed ADOL	OLE	Data cut-off April 21, 2018; median duration (Q1, Q3) of treatment in PED-OLE, 16.6 weeks (8.0, 28.0)	• Dupilumab 300 mg q4w; concomitant TCS and TCI permitted (n = 201) – Patients with IGA score ≥2 after 16 weeks of dupilumab could be up-titrated to 200/300 mg q2w (<60 kg/≥60 kg)
	Children who completed PEDS	OLE	Data cut-off July 22, 2019; median duration (Q1, Q3) of treatment in PED-OLE, 29.6 weeks (18.2, 40.2)	• Dupilumab 300 mg q4w with permitted use of TCS and TCI (n = 335) – Patients with IGA score ≥2 after 16 weeks of dupilumab could be up-titrated to 200/300 mg q2w (<60 kg/≥60 kg)

AD, atopic dermatitis; EASI-75, ≥75% improvement from baseline in Eczema Area and Severity Index; IGA, Investigator’s Global Assessment; n, number of patients in the SAF; OLE, open label; Q1, first quartile; Q3, third quartile; qw, once weekly; q2w, every 2 weeks; q4w, every 4 weeks; q8w, every 8 weeks; RCT, randomized controlled trial; SAF, safety analysis set; SC, subcutaneous; TCI, topical calcineurin inhibitor(s); TCS, topical corticosteroid(s).

^a^The analysis included selected regimens supporting approved posology for pediatric patients aged 6 months to 17 years with AD. For adults, the approved posology is 300 mg q2w, with an initial loading dose of 600 mg. For pediatric patients, the approved posology in the US is as follows: aged 5 months to 5 years, 200 mg q4w for body weight ≥5 to <15 kg and 300 mg q4w for body weight ≥15 to <30 kg; aged 6–17 years, 300 mg q4w (initial loading dose 600 mg) for body weight ≥15 to <30 kg, 200 mg q2w (initial loading dose 400 mg) for body weight ≥30 to <60 kg, and 300 mg q2w (initial loading dose 600 mg) for body weight ≥60 kg (13). In the EU, the approved posology is as follows: aged 6–11 years, 300 mg q4w (loading dose 300 mg at day 1 and 300 mg at day 15) for body weight 15 kg to <60 kg, 300 mg q2w (initial loading dose 600 mg) for body weight ≥60 kg; aged 12–17 years, 300 mg q4w for body weight <60 kg (initial loading dose 400 mg) and 300 mg q2w for body weight ≥60 kg (initial loading dose 600 mg) (13, 14, 36–38).

^b^SOLO-CONTINUE evaluated four dupilumab SC monotherapy regimens vs placebo; dupilumab 300 mg qw and 300 mg q2w were included in this analysis; 300 mg q4w and 300 mg q8w were not included in this analysis.

^c^Dupilumab weight-based dose regimens were pooled for these analyses.

^d^No dupilumab loading dose was administered in this trial.

### 2.2 Ethics

All studies were conducted following the ethical principles derived from the Declaration of Helsinki, the International Conference on Harmonisation guideline, Good Clinical Practice, and local applicable regulatory requirements. Written informed consent was obtained from all patients or their legal guardians prior to commencement of any study procedure.

### 2.3 Assays

#### 2.3.1 Anti-drug antibodies

ADAs in serum samples were assessed using a validated electrochemiluminescence bridging immunoassay, with a mouse anti-dupilumab monoclonal antibody as the positive control and labeled drugs as the bridge components. The assay involved up to three different evaluations: initial screening assay to identify samples potentially positive for ADAs; confirmatory assay to determine whether positive screening assay responses can be inhibited by the presence of excess drug; and a titer procedure to assess ADA levels in confirmed positive samples. Titer values of ADA-positive samples were defined as the maximum final dilution from neat serum that has an assay signal still above the assay cut point (**Table 2**) (**Supplementary Methods: Assays**).

**Table 2:** ADA/NAb status definitions.

Term	Definition
ADA status	
ADA-negative	All samples were negative in the ADA assay
PRE	Positive result at baseline with all post-baseline ADA titers results <4-fold the baseline titer result
TB	Positive result at baseline with at least one post-baseline titer result ≥4-fold the baseline titer result
TE	Negative result or missing result at baseline with at least one positive post-baseline result
In patients with TE-ADA response	
Persistent	Positive ADA result detected in at least two consecutive post-baseline samples separated by ≥12-week post-baseline period with no ADA-negative results in-between
Indeterminate	Positive ADA result at the last collection timepoint only
Transient	Neither persistent nor indeterminate
Maximum ADA titer (only in patients with TE or TB response)^a^	
Low	Titer <1,000
Moderate	Titer ≥1,000 to <10,000
High	Titer ≥10,000
NAb status	
NAb-positive	Positive for NAb assay at ≥1 post-baseline timepoint
NAb-negative	ADA-negative or negative for NAb assay at all timepoints tested

ADA, anti-drug antibody; NAb, neutralizing antibody; PRE, pre-existing immunoreactivity; TB, treatment-boosted; TE, treatment-emergent.

^a^The titer value of an ADA-positive sample was defined as the maximum final dilution from neat serum that had an assay signal still above the assay cut point.

#### 2.3.2 Neutralizing antibodies

The presence of NAbs was evaluated using a validated competitive ligand-binding assay in ADA-positive serum samples. ADA-negative samples were considered NAb-negative (**Supplementary Methods: Assays**).

#### 2.3.3 Dupilumab concentrations in serum

Serum samples for quantitation of dupilumab were analyzed using a validated enzyme-linked immunosorbent assay, with dupilumab as assay standard and human IL-4Rα as capture reagent (**Supplementary Methods: Assays**) (24). Dupilumab concentration was measured as functional antibody with either 1 or 2 free binding sites. The lower limit of quantitation (LLOQ) of functional dupilumab is 0.0780 mg/L in undiluted human serum (25).

### 2.4 Analysis methods

#### 2.4.1 Statistical analyses

All analyses were summarized descriptively and no statistical comparisons were performed. Due to the relatively small number of participants positive for ADAs, individual-level data are presented in figures.

#### 2.4.2 Analysis sets

Safety analysis sets (SAFs) included all patients who received any study drug (dupilumab or placebo). ADA analysis sets included all patients in SAFs with ≥1 non-missing ADA result after the first dose of study drug. NAb analysis sets included all patients in the SAFs with ≥1 non-missing NAb result or who had all samples negative in the ADA assay after the first dose of study drug. PK analysis sets included all randomized patients in the SAFs with ≥1 non-missing drug concentration result following the first dose of study drug. The SAF, ADA, NAb, and PK sets were analyzed according to treatment received.

#### 2.4.3 ADA outcomes

ADA categories included ADA-negative, pre-existing immunoreactivity (PRE), treatment-boosted (TB), and treatment-emergent (TE) (**Table 2**). In SOLO-CONTINUE, OLE, and PED-OLE, parent study baseline was considered baseline for determination of ADA status. TE-ADA responses were further categorized as persistent, indeterminate, or transient (**Table 2**). Maximum post-baseline ADA titer was categorized in TE/TB ADA-positive patients as low (<1,000), moderate (≥1,000 to <10,000), or high (≥10,000), based on maximum titer assay value any time after the first dose. NAb responses were categorized as NAb-positive or NAb-negative. Only ADA-positive samples were tested for NAbs.

#### 2.4.4 Impact of ADAs on PK and efficacy

The impacts of ADAs on PK and efficacy were assessed with scatter plots by nominal time and maximum titer category in ADA-positive patients versus ADA-negative patients. Percent change from baseline in Eczema Area and Severity Index (EASI) was selected as the efficacy endpoint because it is a continuous variable (and thus sensitive to change) and was consistently measured across all clinical trials presented herein.

#### 2.4.5 Impact of ADAs on drug safety

Safety for each trial was summarized by patient ADA status category (ADA-positive, -negative, or missing) using the SAFs. Safety outcomes included treatment-emergent adverse events (TEAEs), TEAEs related to study drug, TEAEs causing permanent discontinuation of study drug, deaths, treatment-emergent serious adverse events (TE-SAEs), TE-SAEs causing permanent discontinuation of study drug, TE-SAEs related to study drug, and certain events of special interest: serum sickness/serum-sickness-like reaction; anaphylaxis/anaphylactic reaction; and Medical Dictionary for Drug Regulatory Activities High Level Term [MedDRA HLT] injection-site reactions (ISRs) lasting >24 hours (note: ADOL, PEDS, and PED-OLE did not collect data for ISRs lasting >24 hours; in the adult OLE, data for ISRs lasting >24 hours were collected only for patients with severe ISRs, and these data were no longer collected after June 2, 2017, when protocol amendment 7 was instituted).

## 3 Results

### 3.1 Patient populations

This analysis included 2,743 patients in 16- or 52-week RCTs (SOLO 1, SOLO 2, ADOL, PEDS, CHRONOS, PRESCHOOL) and 2,536 patients in trials enrolling patients from previous dupilumab studies (SOLO-CONTINUE, OLE, PED-OLE) (**Table 1**). The ADA analysis set included 2,674 patients in the 16- and 52-week RCTs (**Table 3**) and 2,507 patients in trials enrolling patients from previous dupilumab studies (**Supplementary Table E2**).

**Table 3:** Patient ADA/NAb status, persistence, and maximum titer category in randomized, placebo-controlled phase 3 trials in adults, adolescents, and children; ADA analysis set.

	Adults						Pediatric trials								
Trial(s) (treatment period)	Pooled SOLO 1 & 2 (16 weeks)			CHRONOS (52 weeks)			ADOL (aged 12–17 years) (16 weeks)		PEDS (aged 6–11 years) (16 weeks)				PRESCHOOL (aged 6 months to 5 years) (16 weeks)		
ADA category, n (%)	Placebo (n = 427)	Dupilumab 300 mg q2w (n = 447)	Dupilumab 300 mg qw (n = 429)	Placebo + TCS (n = 306)	Dupilumab 300 mg q2w + TCS (n = 105)	Dupilumab 300 mg qw + TCS (n = 308)	Placebo (n = 85)	Dupilumab 200/300 mg q2w (n = 81)	Placebo + TCS (n = 116)	Dupilumab 200 mg q2w + TCS (≥30 kg) (n = 57)	Dupilumab 300 mg q4w + TCS (<30 kg) (n = 56)	Dupilumab 300 mg q4w + TCS (any wt)^a^ (n = 114)	Placebo + TCS (n = 69)	Dupilumab 200 mg q4w + TCS (≥5 to <15 kg) (n = 24)	Dupilumab 300 mg q4w + TCS (≥15 to <30 kg) (n = 50)
PRE	16 (3.7)	28 (6.3)	19 (4.4)	18 (5.9)	3 (2.9)	18 (5.8)	4 (4.7)	1 (1.2)	3 (2.6)	1 (1.8)	2 (3.6)	4 (3.5)	2 (2.9)	0	0
TB response	2 (0.5)	0	0	1 (0.3)	1 (1.0)	1 (0.3)	0	0	0	0	0	0	0	0	0
TE response	6 (1.4)	33 (7.4)	12 (2.8)	23 (7.5)	9 (8.6)	18 (5.8)	3 (3.5)	13 (16.0)	2 (1.7)	3 (5.3)	0	0	0	0	1 (2.0)
Persistent	0	1 (0.2)	1 (0.2)	9 (2.9)	2 (1.9)	4 (1.3)	1 (1.2)	3 (3.7)	0	0	0	0	0	0	0
Transient	0	3 (0.7)	0	8 (2.6)	5 (4.8)	9 (2.9)	1 (1.2)	9 (11.1)	1 (0.9)	2 (3.5)	0	0	0	0	0
Indeterminate	6 (1.4)	29 (6.5)	11 (2.6)	6 (2.0)	2 (1.9)	5 (1.6)	1 (1.2)	1 (1.2)	1 (0.9)	1 (1.8)	0	0	0	0	1 (2.0)
ADA-negative^b^	403 (94.4)	386 (86.4)	398 (92.8)	264 (86.3)	92 (87.6)	271 (88.0)	78 (91.8)	67 (82.7)	111 (95.7)	53 (93.0)	54 (96.4)	110 (96.5)	67 (97.1)	24 (100)	49 (98.0)
Maximum ADA titer^c^															
Low (<1,000)	8 (1.9)	30 (6.7)	7 (1.6)	24 (7.8)	9 (8.6)	17 (5.5)	3 (3.5)	11 (13.6)	2 (1.7)	3 (5.3)	0	0	0	0	1 (2.0)
Moderate (≥1,000 and ≤10,000)	0	3 (0.7)	3 (0.7)	0	1 (1.0)	2 (0.6)	0	2 (2.5)	0	0	0	0	0	0	0
High (>10,000)	0	0	2 (0.5)	0	0	0	0	0	0	0	0	0	0	0	0
**NAb analysis set**	427 (100)	447 (100)	429 (100)	306 (100)	105 (100)	308 (100)	85 (100)	81 (100)	116 (100)	57 (100)	56 (100)	114 (100)	69 (100)	24 (100)	50 (100)
NAb-negative	425 (99.5)	436 (97.5)	425 (99.1)	303 (99.0)	104 (99.0)	307 (99.7)	84 (98.8)	77 (95.1)	116 (100)	56 (98.2)	56 (100)	114 (100)	69 (100)	24 (100)	50 (100)
NAb-positive	2 (0.5)	11 (2.5)	4 (0.9)	3 (1.0)	1 (1.0)	1 (0.3)	1 (1.2)	4 (4.9)	0	1 (1.8)	0	0	0	0	0

ADA, anti-dupilumab antibody; NAb, neutralizing antibody; PRE, pre-existing immunogenicity detected at parent study baseline; qw, once weekly; q2w, every 2 weeks; q4w, every 4 weeks; TB, treatment-boosted; TCS, topical corticosteroid(s); TE, treatment-emergent.

^a^Approved EU dose regimen (14).

^b^ADA-negative: all samples were negative in the ADA assay.

^c^Maximum titer category is reported only for patients who were ADA-positive at any time. The titer value of an ADA-positive sample is defined as the maximum dilution of the sample that has an assay signal still above the assay cut point.

### 3.2 ADA status

#### 3.2.1 ADA incidence

Most dupilumab-treated patients were ADA-negative (dupilumab: 82.7–100.0%; placebo: 83.8–97.1%) (**Table 3**). In the RCTs, TE-ADA responses were observed in up to 8.6% (adults), 16.0% (adolescents), 5.3% (children), and 2.0% (preschoolers/infants) dupilumab-treated patients; and 0–7.5% placebo-treated patients (**Table 3**). Incidence of TE-ADA responses with dupilumab treatment was similar in the long-term trials that enrolled patients from previous trials (**Supplementary Table E2**). In SOLO-CONTINUE, incidence of TE-ADA was higher for patients who switched from dupilumab to placebo (11.3%) than those who continued on dupilumab (2.9%) (**Supplementary Table E2**). A small number of patients exhibited pre-existing immunoreactivity at baseline in the RCTs (dupilumab: 1.2–6.3%; placebo: 2.6–5.9%); among these patients, TB responses were uncommon (≤1% of placebo-treated or dupilumab-treated patients) (**Table 3**).

#### 3.2.2 ADA persistence

Incidence of persistent TE-ADA responses was low. Among dupilumab-treated patients, persistent TE-ADA responses were observed in 0.2–1.9% of adults, 3.7% of adolescents, and no children in the RCTs (**Table 3**). In the trials that enrolled patients from previous trials, persistent TE-ADA responses were observed in no patients in SOLO-CONTINUE, 0.9% of patients in the adult OLE, and 1.5% adolescents and 0.6% children in PED-OLE (**Supplementary Table E2**). In the adult OLE, 4.3% of TE-ADA responses were transient and 0.9% were persistent; most ADA-positive patients became ADA-negative by week 20–44 and remained ADA-negative through the 3 years included in this analysis (data not shown).

#### 3.2.3 ADA titers

**Figure 1:**
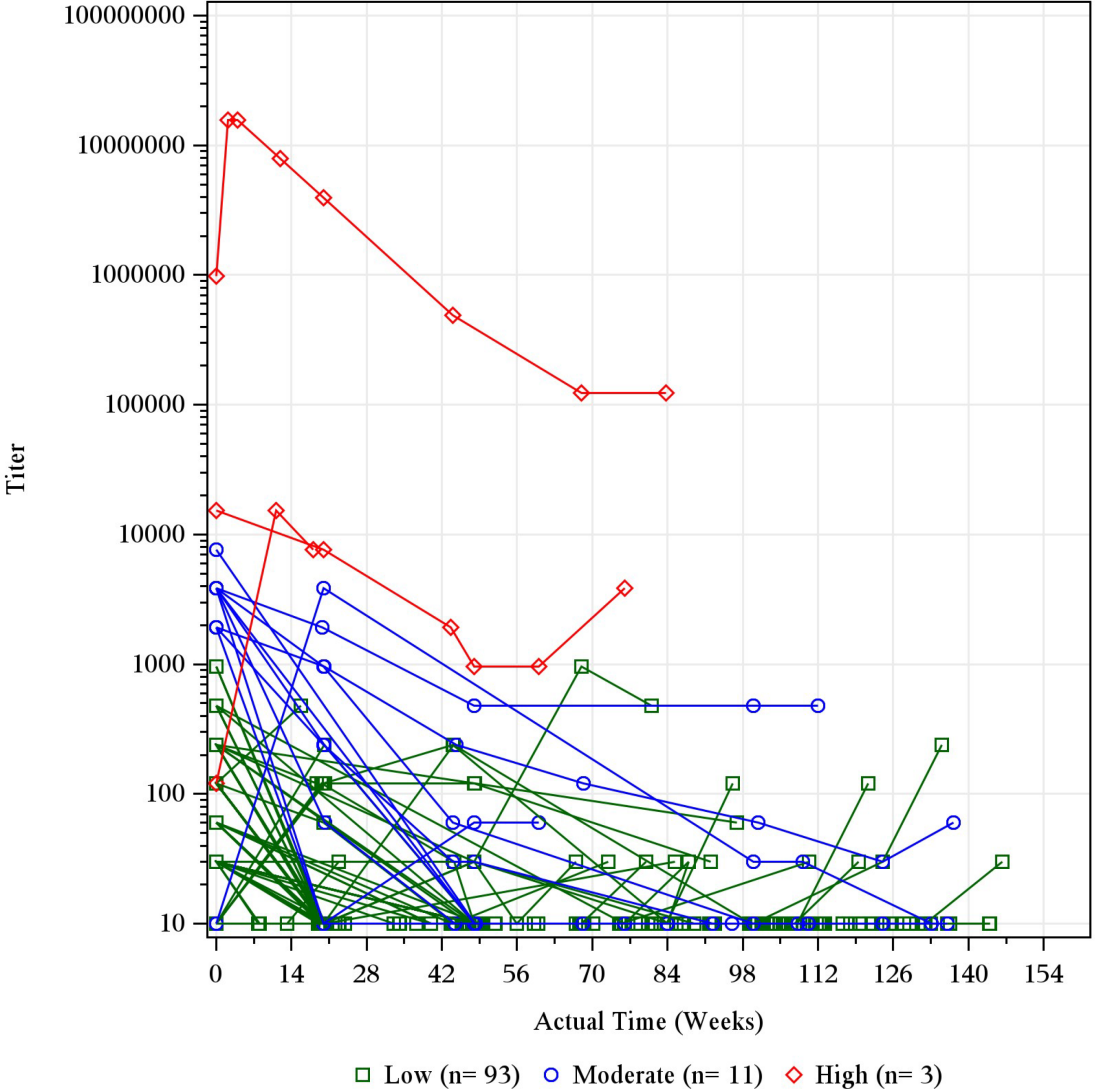
Individual titer over time by maximum titer category in ADA-positive patients in the adult OLE. Baseline records from parent studies were used to derive maximum titer category and ADA status. The titer was imputed as 10 for ADA-negative results for the purpose of this figure. ADA, anti-drug antibody; n, number of participants with titer category; OLE, open-label extension; qw, once weekly.

Most ADA-positive dupilumab-treated patients exhibited a low (<1,000) maximum post-baseline ADA titer, while high titers (≥10,000) were rare (<1% of dupilumab-treated patients) (**Table 3**; **Supplementary Table E2**). In the adult OLE, three patients had high-titer responses and 11 had moderate titers (**Supplementary Table E2**); in nearly all these patients, titers declined over time with continued dupilumab dosing (**Figure 1**).

Among placebo-treated patients with ADA-positive responses, nearly all exhibited low titers; only one (in SOLO-CONTINUE) exhibited a moderate titer (**Table 3**; **Supplementary Table E2**).

### 3.3 NAbs

Across all trials, most dupilumab-treated patients (91.3–100%) were NAb-negative (**Table 3**; **Supplementary Table E2**). In the 16-week RCTs, among dupilumab-treated patients, NAbs were observed in up to 2.5% adults, 4.9% adolescents, 1.8% children, and no preschoolers/infants; among placebo-treated patients, NAbs were observed in 0.5% adults, 1.2% adolescents, and no children or preschoolers/infants (**Table 3**). In long-term trials (i.e. with duration ≥36 weeks), NAb incidence was similarly low. Among dupilumab-treated patients, NAbs were observed in 0.3–1.0% of patients in CHRONOS, 0–1.2% in SOLO-CONTINUE, 2.6% in the adult OLE, and 5.1% of adolescents and no children in PED-OLE. Among placebo-treated patients, NAbs were observed in 1.0% of patients in CHRONOS and 5.0% in SOLO-CONTINUE (**Table 3**; **Supplementary Table E3**). Across all studies and treatment groups, dupilumab-treated patients with moderate- or high-titer ADAs were more likely to also be NAb-positive compared with patients with low-titer ADAs (**Table 4**; **Supplementary Figure E1**).

**Table 4:** Proportion of patients pooled from SOLOs, CHRONOS, ADOL, PEDS, and PRESCHOOL with TE or TB ADAs that were NAb-positive, by maximum titer category.

Maximum ADA titer category (TE & TB)	Proportion of NAb- positive patients, n/N (%)
Low (<1,000)	23/135 (17.04)
Moderate (≥1,000 and ≤10,000)	7/11 (63.64)
High (>10,000)	2/2 (100.0)

ADA, anti-dupilumab antibody; n, number of NAb-positive patients in titer category; N, number of patients in titer category; NAb, neutralizing antibody; TB, treatment-boosted; TE, treatment-emergent.

### 3.4 Impact of ADAs on functional concentration of dupilumab in serum

**Figure 2:**
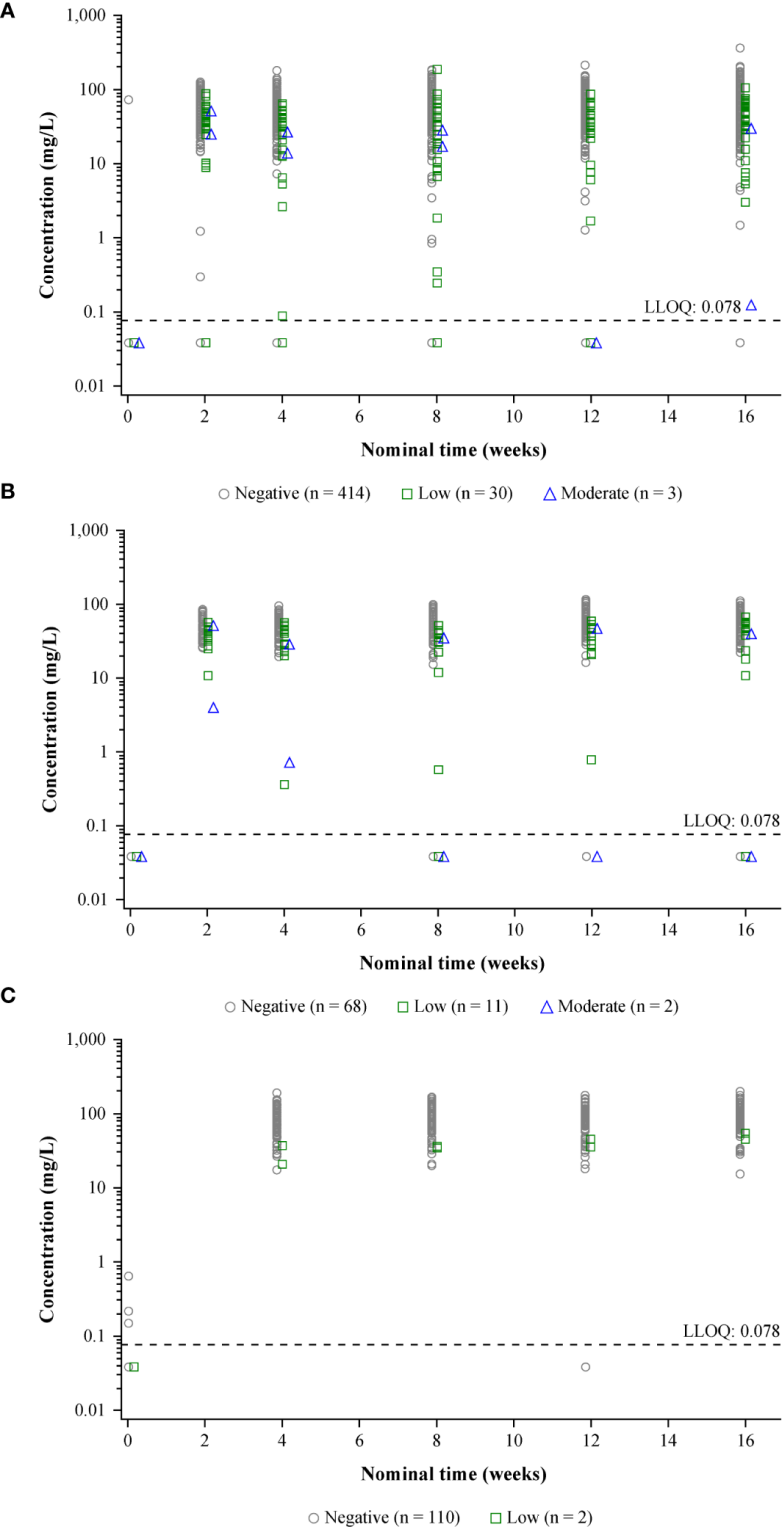
Individual concentrations of functional dupilumab in serum by nominal time and maximum ADA titer category.^a^ **(A)** Pooled SOLO 1 and 2: dupilumab 300 mg q2w. **(B)** ADOL: dupilumab 200/300 mg q2w. **(C)** PEDS: dupilumab 200 mg q2w + TCS (≥30 kg)/300 mg q4w + TCS (<30 kg). Concentrations below the LLOQ (horizontal dashed line) were set to LLOQ/2. Concentration results are jittered by maximum titer category on the X-axis for better data presentation. ADA, anti-drug antibody; LLOQ, lower limit of quantitation; q2w, every 2 weeks; q4w, every 4 weeks; TCS, topical corticosteroid(s). ^a^Low = ADA titer <1,000; moderate = ADA titer ≥1,000 to ≤10,000; high = ADA titer >10,000.

Functional concentrations of dupilumab in serum in ADA-positive patients generally fell within the range of concentrations for ADA-negative patients. For patients with low titers, dupilumab concentrations were generally within the range observed in ADA-negative patients. However, for a small number of cases in patients with high (and, infrequently, moderate) titers, concentrations were lower than in ADA-negative patients (**Figure 2**; **Supplementary Figure E2**) – in these patients, the concentrations of dupilumab were below the LLOQ at most timepoints, which is considered as essentially non-detectable. In two of the three patients with high-titer ADAs in the adult OLE, dupilumab concentration in serum remained low, while ADA titers decreased over time; in contrast, in patients with moderate-titer ADAs, concentrations of dupilumab tended to increase as titer decreased over time (**Supplementary Figure E3**).

### 3.5 Impact of ADAs on efficacy

**Figure 3:**
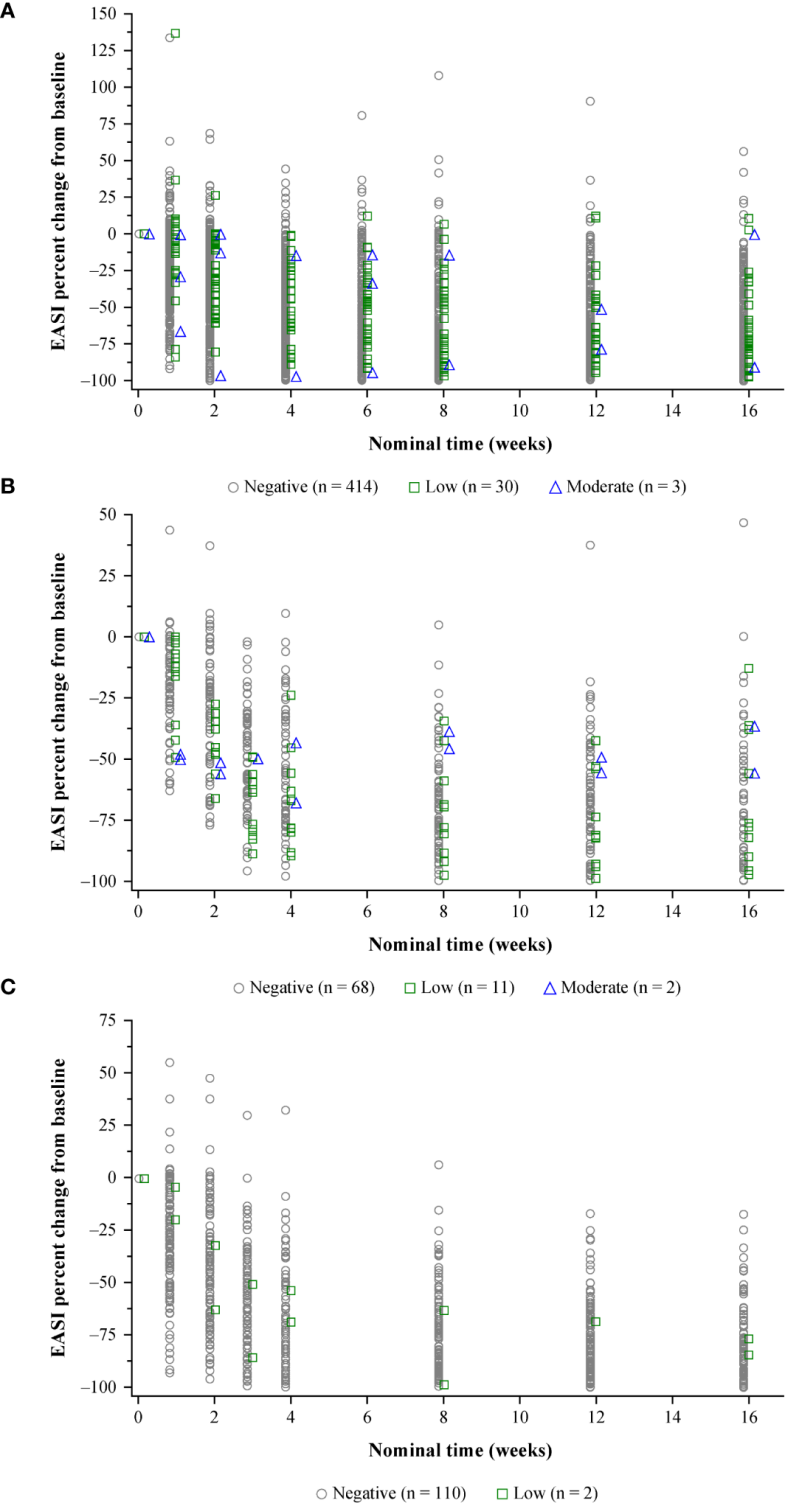
Efficacy (percent change in EASI from baseline) by nominal time and maximum ADA titer category.^a^ **(A)** Pooled SOLO 1 and 2: dupilumab 300 mg q2w. **(B)** ADOL: dupilumab 200/300 mg q2w. **(C)** PEDS: dupilumab 200 mg q2w + TCS (≥30 kg)/300 mg q4w + TCS (≤30 kg). Results are jittered by maximum titer category on the X-axis for better data presentation. ADA, anti-dupilumab antibody; EASI, Eczema Area and Severity Index; q2w, every 2 weeks; q4w, every 4 weeks; TCS, topical corticosteroid(s). ^a^Low = ADA titer <1,000; moderate = ADA titer ≥1,000 to ≤10,000; high = ADA titer >10,000.

In the RCTs, the few adult and pediatric patients with high-titer ADA responses were less likely to exhibit sustained improvement (decreases) in EASI scores from baseline than patients with low-titer or ADA-negative responses (**Supplementary Figure E4A**). The impact of moderate-titer responses in the RCTs was variable, with some patients exhibiting near-complete resolution of AD, while others demonstrated negligible improvement in EASI over the study (**Figure 3**; **Supplementary Figure E4**). For patients with low titers, improvement in EASI from baseline fell within the range of improvement reported for ADA-negative patients (**Figure 3**; **Supplementary Figure E4**).

Few patients in the OLEs had moderate- or high-titer responses (**Supplementary Table E2**). With continued dupilumab treatment in the adult OLE, ADA titer decreased and concurrent improvements in efficacy were observed (**Supplementary Figure E3**). Two patients in PED-OLE had high-titer responses; one showed no improvement in EASI, while the other showed moderate improvement (**Supplementary Table E2**; **Supplementary Figures E4F–I**). Efficacy was unaffected by low-titer ADAs in PED-OLE (**Supplementary Figures E4F–I**).

### 3.6 Impact of ADAs on drug safety

#### 3.6.1 Overview

The proportions of patients with ≥1 TEAE were similar between ADA-negative and ADA-positive patients (**Table 5**; **Supplementary Table E3**). There was no clear relationship between ADA status and incidence of TEAEs considered by the investigators to be related to study drug, TEAEs leading to permanent discontinuation of study drug, or TE-SAEs. In addition, there was no clear relationship between ADA titer and TE-SAEs in any of the trials, with the caveat that this is limited by lower n in the high- and moderate-titer groups (**Supplementary Table E4**).

**Table 5:** Safety outcomes in randomized, placebo-controlled phase 3 trials. (A) Adults. (B) Adolescents, children, and infants.

(A) Adults.							
Number of patients with at least one such event, n1/N (%)	ADA status^a^	Adult trials					
		SOLO 1 & 2 pooled (16 weeks)			CHRONOS (52 weeks)		
		Placebo (n = 456)	Dupilumab 300 mg q2w (n = 465)	Dupilumab 300 mg qw (n = 455)	Placebo + TCS (n = 315)	Dupilumab 300 mg q2w + TCS (n = 110)	Dupilumab 300 mg qw + TCS (n = 315)
Overview							
Any TEAE	ADA+	8/8 (100)	23/33 (69.7)	10/12 (83.3)	21/24 (87.5)	9/10 (90.0)	18/19 (94.7)
	ADA−	290/419 (69.2)	290/414 (70.0)	281/417 (67.4)	240/282 (85.1)	84/95 (88.4)	243/289 (84.1)
	Missing	15/29 (51.7)	8/18 (44.4)	16/26 (61.5)	7/9 (77.8)	4/5 (80.0)	2/7 (28.6)
TEAE related to study drug	ADA+	3/8 (37.5)	12/33 (36.4)	5/12 (41.7)	7/24 (29.2)	2/10 (20.0)	7/19 (36.8)
	ADA−	82/419 (19.6)	116/414 (28.0)	123/417 (29.5)	83/282 (29.4)	35/95 (36.8)	102/289 (35.3)
	Missing	4/29 (13.8)	2/18 (11.1)	6/26 (23.1)	2/9 (22.2)	0	1/7 (14.3)
TEAE causing permanent discontinuation of study drug	ADA+	1/8 (12.5)	0	1/12 (8.3)	1/24 (4.2)	0	3/19 (15.8)
	ADA−	2/419 (0.5)	5/414 (1.2)	3/417 (0.7)	23/282 (8.2)	1/95 (1.1)	5/289 (1.7)
	Missing	4/29 (13.8)	1/18 (5.6)	3/26 (11.5)	1/9 (11.1)	1/5 (20.0)	1/7 (14.3)
Death	ADA+	0	0	0	0	0	0
	ADA−	0	0	0	0	0	1/289 (0.3)
	Missing	0	0	1/26 (3.8)	0	0	0
Any TE-SAE	ADA+	0	0	0	0	0	2/19 (10.5)^b^
	ADA−	20/419 (4.8)	10/414 (2.4)	6/417 (1.4)	16/282 (5.7)	3/95 (3.2)	7/289 (2.4)
	Missing	4/29 (13.8)	1/18 (5.6)	4/26 (15.4)	0	1/5 (20.0)	1/7 (14.3)
TE-SAE related to study drug	ADA+	0	0	0	0	0	2/19 (10.5)^b^
	ADA−	3/419 (0.7)	2/414 (0.5)	1/417 (0.2)	3/282 (1.1)	1/95 (1.1)	0
	Missing	0	0	1/26 (3.8)	0	0	0
TE-SAE causing permanent discontinuation of study drug	ADA+	0	0	0	0	0	2/19 (10.5)^b^
	ADA−	2/419 (0.5)	2/414 (0.5)	0	2/282 (0.7)	0	0
	Missing	4/29 (13.8)	1/18 (5.6)	2/26 (7.7)	0	0	1/7 (14.3)
Adverse events of special interest							
Serum sickness/serum-sickness-like reaction^c^	ADA+	0	0	0	0	0	0
	ADA−	0	0	0	0	0	0
	Missing	0	0	0	0	0	0
Anaphylaxis/anaphylactic reaction MedDRA SMQ narrow^d^	ADA+	0	0	0	0	0	0
	ADA−	0	0	0	0	0	0
	Missing	0	0	0	0	0	0
ISRs lasting >24 hours^e^	ADA+	0	0	0	0	0	0
	ADA−	0	0	0	0	0	0
	Missing	0	0	0	0	0	0

(B) Adolescents, children, and infants.									
Number of patients with at least one such event, n1/N (%)	ADA status^a^	Pediatric trials							
		ADOL (aged 12–17 years) (16 weeks)		PEDS (aged 6–11 years) (16 weeks)				PRESCHOOL (aged 6 months to 5 years) (16 weeks)	
		Placebo (n = 85)	Dupilumab 200/300 mg q2w (n = 82)	Placebo + TCS (n = 120)	Dupilumab 200 mg q2w + TCS (≥30 kg) (n = 59)	Dupilumab 300 mg q4w + TCS (<30 kg) (n = 60)	Dupilumab 300 mg q4w + TCS (any wt)^f^ (n = 120)	Placebo+ TCS (n = 78)	Dupilumab 200/300 mg q4w + TCS (n = 83)
Overview									
Any TEAE	ADA+	3/3 (100)	9/13 (69.2)	2/2 (100)	1/3 (33.3)	0/0 (0)	0/0 (0)	0/0 (0)	1/1 (100)
	ADA−	57/82 (69.5)	49/68 (72.1)	83/114 (72.8)	35/54 (64.8)	37/56 (66.1)	75/114 (65.8)	52/69 (75.4)	49/73 (67.1)
	Missing	0/0 (0)	1/1 (100)	3/4 (75.0)	0/2 (0)	2/4 (50.0)	3/6 (50.0)	6/9 (66.7)	3/9 (33.3)
TEAE related to study drug	ADA+	1/3 (33.3)	3/13 (23.1)	0	1/3 (33.3)	0	0	0	0
	ADA−	12/82 (14.6)	14/68 (20.6)	13/114 (11.4)	11/54 (20.4)	13/56 (23.2)	23/114 (20.2)	3/69 (4.3)	9/73 (12.3)
	Missing	0	1/1 (100)	0	0	0	1/6 (16.7)	2/9 (22.2)	0
TEAE causing permanent discontinuation of study drug	ADA+	0	0	0	0	0	0	0	0
	ADA−	1/82 (1.2)	0	2/114 (1.8)	1/54 (1.9)	0	0	0	1/73 (1.4)
	Missing	0	0	0	0	0	0	1/9 (11.1)	0
Death	ADA+	0	0	0	0	0	0	0	0
	ADA−	0	0	0	0	0	0	0	0
	Missing	0	0	0	0	0	0	0	0
Any TE-SAE	ADA+	0	0	0	0	0	0	0	0
	ADA−	1/82 (1.2)	0	2/114 (1.8)	0	2/56 (3.6)	2/114 (1.8)	3/69 (4.3)	0
	Missing	0	0	0	0	0	0	1/9 (11.1)	0
TE-SAE related to study drug	ADA+	0	0	0	0	0	0	0	0
	ADA−	0	0	0	0	0	0	0	0
	Missing	0	0	0	0	0	0	0	0
TE-SAE causing permanent discontinuation of study drug	ADA+	0	0	0	0	0	0	0	0
	ADA−	0	0	0	0	0	0	0	0
	Missing	0	0	0	0	0	0	0	0
Adverse events of special interest									
Serum sickness/serum-sickness-like reaction^c^	ADA+	0	0	0	0	0	0	0	0
	ADA−	0	0	0	0	0	0	0	0
	Missing	0	0	0	0	0	0	0	0
Anaphylaxis/anaphylactic reaction MedDRA SMQ narrow^d^	ADA+	0	0	0	0	0	0	0	0
	ADA−	0	0	0	0	0	0	0	0
	Missing	0	0	0	0	0	0	0	0
ISRs lasting >24 hours^e^	ADA+	n/a	n/a	n/a	n/a	n/a	n/a	0	0
	ADA−	n/a	n/a	n/a	n/a	n/a	n/a	0	0
	Missing	n/a	n/a	n/a	n/a	n/a	n/a	0	0

ADA, anti-drug antibody; ADA−, ADA-negative; ADA+, ADA-positive; HLT, MedDRA High Level Term; ISR, injection-site reaction; MedDRA, Medical Dictionary for Regulatory Activities, N, number of patients in the ADA group for each dose group; n, number of patients in dose group, based on the safety analysis set; n1, number of patients with an event; n/a, not available; PRE, pre-existing immunoreactivity; PT, MedDRA Preferred Term; qw, weekly; q2w, every 2 weeks; q4w, every 4 weeks; SMQ, standardized MedDRA query; TB, treatment-boosted; TCS, topical corticosteroid(s); TE, treatment-emergent; TEAE, treatment-emergent adverse event; TE-SAE, treatment-emergent serious adverse event; wt, weight.

^a^For the purpose of the safety analysis, ADA+ included TE or TB, and ADA− included either negative at all timepoints or PRE.

^b^The two TE-SAEs in the ADA-positive group for dupilumab 300 mg qw in CHRONOS were cystoid macular edema and rash maculo-papular (MedDRA PTs); both events led to permanent discontinuation of study drug.

^c^MedDRA PTs.

^d^MedDRA PTs included in the MedDRA “anaphylaxis/anaphylactic reaction SMQ narrow”.

^e^MedDRA HLT. ISRs lasting >24 h were not defined in ADOL or PEDS.

^f^Approved dose regimen in EU.

#### 3.6.2 Adverse events of special interest

One event of serum sickness was reported, in a woman with high-titer ADAs in the adult OLE (**Supplementary Appendix** [patient narratives]). The event occurred on day 18, after her second dose of dupilumab; it resolved by day 21, and was considered by the investigator to be related to study drug. She permanently discontinued study drug after this event. She had received placebo in SOLO 1 and SOLO-CONTINUE, and consistently exhibited a pre-existing low-titer ADA response throughout SOLO 1 and SOLO-CONTINUE (titer range 120–480). Her ADA titer was 120 on day 0 of OLE, increased to 15,360 at the end of treatment (early termination visit, at approximately 79 days), and then decreased to 7,680 at the end of study visit at day 129. She also tested NAb-positive throughout OLE.

No events of anaphylaxis were reported in ADA-positive patients. All anaphylaxis/anaphylactic-type events occurred in patients with known food allergies, after a minimum of 5 doses of dupilumab, and none were considered related to study drug. These events were rare and were only reported in 1/83 (1.2%) ADA-negative patients in the dupilumab 300 mg weekly (qw) group in SOLO-CONTINUE, 4/1,644 (0.2%) ADA-negative patients in the dupilumab 300 mg qw group in OLE, and 3/318 (0.9%) ADA-negative patients in PEDS-OLE.

No patients in any studies included in this analysis reported ISRs lasting >24 hours (these data were not collected in ADOL, PEDS, or PED-OLE) (**Table 5**; **Supplementary Table E3**).

## 4 Discussion

This analysis includes ~3,000 patients with moderate-to-severe AD across multiple phase 3 RCTs and OLEs (15–23). The RCTs assessed immunogenicity in a controlled setting for 16 or 52 weeks. Dupilumab is intended for chronic administration; hence the OLE studies allowed for longitudinal assessment of immunogenicity, starting from the parent study, for up to 4 years of dupilumab treatment for adults and up to 68 weeks for pediatric patients. Most participants treated with dupilumab did not develop ADAs. Among those few who developed ADAs, titers were generally low (<1,000) and transient, and most patients were NAb-negative. A smaller subset (≤2.5%) developed moderate titers (≥1,000 and ≤10,000) and very few (<1%) developed high titers (>10,000). Immunogenic responses had minimal impact on dupilumab concentration, efficacy, and safety, except for the very few patients with high-titer ADAs.

ADA incidence and distribution of patients among maximum titer categories in dupilumab-treated patients were generally similar across studies, age groups, and dose regimens. TE-ADA incidence rates were higher for dupilumab-treated adolescents in ADOL (16%) and PED-OLE (12.2%) than for adults (2.8–8.6%) or younger age groups (0–5.3%). This could be explained by the mostly transient ADAs assessed at week 4 in ADOL, reflective of an IgM rather than an IgG ADA response (26). The ADA assay detects both IgM and IgG responses. Many IgM responses are transient and do not class switch to the more durable IgG (25). There is also a low false positivity rate in the assay, as demonstrated by the low TE-ADA incidence rates observed in the placebo-treated patients.

TE-ADA incidence by age group was generally similar in the RCTs and OLEs, suggesting that longer-term treatment did not affect overall incidence rates (however, withdrawal of patients over time in the OLE may affect this outcome). Indeed, patients who develop an ADA response are most likely to do so at the onset of treatment, and few ADA responses were persistent.

Overall, immunogenicity had minimal effect on drug concentrations and efficacy of dupilumab. The range of dupilumab concentrations and treatment efficacy in patients with low-titer ADAs was generally within the distribution for ADA-negative patients. High-titer ADAs, while rare, were more likely to be associated with lower concentrations of dupilumab and diminished improvement in EASI. In patients with moderate-titer ADAs, both systemic dupilumab concentrations in serum and efficacy fell between the low- and high-titer responses, supporting appropriateness of the titer thresholds associated with the categories. Influence of moderate- or high-titer responses on efficacy was more pronounced in 16-week RCTs than OLEs, in which the range of improvements in EASI appeared independent of titer. With continued dosing in the adult OLE, dupilumab serum concentrations and efficacy generally improved over time as ADA titers declined; decreases were most prominent in patients with moderate or high titers. These patterns suggest that patients may continue to benefit from ongoing treatment with dupilumab irrespective of their ADA titer.

Approximately 80% of patients who developed ADAs were NAb-negative. Although NAb status was correlated with higher ADA titers, maximum titer category (e.g. low-/moderate-/high-titer) was a more discriminating determinant to assess the impact of immunogenicity on dupilumab PK, efficacy, and safety than any other metric, including NAb-positivity. Importantly, in the adult OLE, there was no increase in ADA incidence, persistence, or NAbs over long-term treatment; incidence rates were generally similar to those observed in the shorter-duration RCTs. In the PED-OLE, ADA incidence, persistence, or NAbs were also generally similar compared with the 16-week pediatric RCTs. It should be noted that ADA specificity is not a purely stochastic process, and the role of immunodominant epitopes is widely appreciated; the assays performed in this study did not discriminate between epitopes or binding strength. Correlations in incidence between ADA titer level and incidence of NAbs for dupilumab may not be generalizable to other therapeutic proteins.

Three adverse events of special interest were evaluated in this analysis: serum sickness/serum-sickness-like reactions; anaphylaxis/anaphylactic reactions; and ISRs lasting >24 hours. In this analysis, serum sickness was reported in one patient with a high-titer ADA response and NAb-positivity after 2 doses of dupilumab in the adult OLE; this event resolved 3 days after it was reported (**Supplementary Appendix** [patient narratives]). This patient was previously treated with placebo and had pre-existing low ADA titers during both parent studies prior to OLE. The event was considered by the investigator to be related to study drug, and study drug was permanently discontinued. Only two other events of serum sickness have been reported in dupilumab clinical trials in AD, both in RCTs not included in this analysis: LIBERTY AD CAFÉ [NCT02755649] and LIBERTY AD EVALUATE [NCT022107080] (summarized in the **Supplementary Appendix** [patient narratives]) (13, 14, 27). The patient in EVALUATE exhibited a high-titer ADA response; in contrast, the patient in CAFÉ had a low-titer response, and had symptoms atypical for serum sickness (13, 14, 27). All three cases of serum sickness occurred early in dupilumab treatment, after 1 to 3 doses of dupilumab; all events resolved and were considered related to study drug; and all patients permanently discontinued study drug. Despite the paucity of data, it seems possible that the occurrence of serum sickness, while rare, could be associated with high-titer ADAs.

None of the cases of anaphylaxis/anaphylactic reactions were ADA-positive; these cases were all related to known food allergies, and none were considered related to study drug.

No patients reported an ISR lasting >24 hours (note that ADOL, PEDS, and PED-OLE did not collect these data, and the adult OLE only collected these data for patients with severe ISRs); thus, in this analysis showed no evidence of any relationship between this event and immunogenicity.

The incidence of TEAEs were similar for ADA-positive and ADA-negative patients. For the approved dose regimens, serious TEAEs were less frequent in ADA-positive than ADA-negative patients. Except in the rare instances of patients with high-titer ADAs, there was no clear association between ADA status or titers (moderate/low) and relative incidence rates of drug-related TEAEs and permanent discontinuations due to TEAEs.

The ADA assay used in these trials was highly drug tolerant; detection of binding anti-dupilumab responses would not be limited by trough drug concentrations at the studied doses. The NAb assay had a substantially lower drug tolerance (**Supplementary Methods: Assays**), such that trough drug concentrations could interfere with NAb detection in ADA-positive samples. The lack of NAbs in low-titer ADA responses may indicate targeting of other epitopes on dupilumab or be due to the technical limitations of the assay. However, the functional dupilumab PK assay uses a target capture format; presence of NAbs at sufficient levels would inhibit drug capture on the plate and result in reduced concentrations (28). The fact that similar drug concentrations were observed in both ADA-negative and low- to moderate-titer ADA-positive patients indicates that any neutralizing responses undetected in the NAb assay were not clinically impactful.

While all therapeutic proteins may induce immunogenicity, the propensity to develop ADAs and the impact thereof can vary widely. For example, monoclonal antibodies directed against tumor necrosis factor (TNF)-α have shown immunogenicity incidence rates ranging from 5% to 67% of patients, with significant impact on drug concentrations, efficacy, and safety (2, 7, 8, 29–32); other monoclonal antibodies (e.g. anti-IL-12/13, anti-IL-6, anti-IL-17) appear to generate lower immunogenicity levels (e.g. <10%), with little or no clinical consequence (8, 33–36). However, caution should be used when comparing ADA responses between drugs due to multiple factors that could influence immunogenicity assessment, including assay sensitivities, sampling schedules, treatment regimens, monoclonal antibody humanization, target molecules, and disease immunoreactivity (8).

A strength of this analysis is inclusion of a large population of dupilumab-treated patients spanning age groups from infancy to adulthood, and the consistency of PK, ADA, and NAb assays across all trials. Another advantage is that we assessed immunogenicity in both RCTs and longitudinal OLEs. The RCTs assessed immunogenicity in a controlled setting for 16 or 52 weeks. Of note, although the current analysis is focused on AD, immunogenicity incidence is low across all other indications (13, 14). A limitation of this analysis is that longitudinal data on changes in ADA status for individual patients were not assessed over time at the patient level, except for assessment of titer over time in the adult OLE. Patients were considered ADA-positive if they had any TE or TB responses, regardless of number of positive samples or trial length. Although we were able to identify whether ADA-positive results were persistent or transient within a study, the full time-course of ADA-positivity was not evaluated. Finally, due to the limited ADA sampling frequency in these studies, in most cases no temporal correlation can be made between ADA-positivity and TEAE incidence.

## 5 Conclusions

In conclusion, across adults and pediatric patients (including infants), most patients with moderate-to-severe AD treated with dupilumab did not develop ADAs. Among those who developed ADAs, titers were generally low, transient, and mostly NAb-negative. Minimal to no impact of ADAs on dupilumab concentration, efficacy, and safety was observed, except for some rare instances of high-titer responses. In rare cases, serum sickness/serum-sickness-like reactions have been observed in some patients with high-titer ADAs. The longitudinal analysis suggests that patients with ADA responses may benefit from continued use of dupilumab.

## Data Availability

Qualified researchers may request access to study documents (including the clinical study report, study protocol with any amendments, blank case report form, statistical analysis plan) that support the methods and findings reported in this manuscript. Individual anonymized participant data will be considered for sharing 1) once the product and indication has been approved by major health authorities (e.g. FDA, EMA, PMDA, etc) or development of the product has been discontinued globally for all indications on or after April 2020 and there are no plans for future development 2) if there is legal authority to share the data and 3) there is not a reasonable likelihood of participant re-identification. Submit requests to https://vivli.org/. Requests to access the datasets should be directed to Mohamed Kamal, mohamed.kamal@regeneron.com.
