# Supplementary material for: Immunogenicity of dupilumab in adult and pediatric patients with atopic dermatitis
Source: Front Immunol. 2024 Nov 11;15:1466372. doi: 10.3389/fimmu.2024.1466372 (PMC11586717; doi:10.3389/fimmu.2024.1466372)
Supplement: Supplementary file 1 [file DataSheet1.docx]

**Supplementary appendix: Immunogenicity of dupilumab in adult and pediatric patients with atopic dermatitis**

Contents

[Supplementary Methods: Assays 4](#_Toc175919593)

[Patient narratives of serum sickness or serum sickness-like reactions 6](#_Toc175919594)

[TABLE E1. Blood sample timepoints for ADAs and PK assessment 8](#_Toc175919595)

[TABLE E2. Patient ADA/NAb status and maximum titer category for long-term trials: SOLO-CONTINUE, OLE, and PED-OLE; ADA analysis set 9](#_Toc175919596)

[TABLE E3. Safety outcomes for long-term trials: SOLO-CONTINUE, OLE, and PED-OLE 11](#_Toc175919597)

[TABLE E4. Safety outcomes (TE-SAEs and permanent discontinuation of study drug) by ADA titer (high, moderate, low). A. RCTs; B. Long-term trials 14](#_Toc175919598)

[FIG E1. Proportion of patients pooled from SOLOs, CHRONOS, ADOL, PEDS, and PRESCHOOL with TE or TB ADAs that were NAb-positive, by maximum titer category. 17](#_Toc175919599)

[FIG E2. Individual concentrations of functional dupilumab in serum by nominal time and maximum ADA titer category.^a^ 18](#_Toc175919602)

[FIG E3. Individual log-scaled concentration of functional dupilumab in serum and EASI percent change from baseline over time by ADA titer in patients with high- or moderate-titer ADAs in the adult OLE 21](#_Toc175919603)

[FIG E4. Efficacy: percent change in EASI from baseline by nominal time and maximum ADA titer category. 22](#_Toc175919604)

[Supplementary references 25](#_Toc175919605)

# Supplementary Methods: Assays

**Anti-Drug Antibodies (ADAs).** ADAs in serum samples were assessed using a validated electrochemiluminescence bridging immunoassay, with a mouse anti-dupilumab monoclonal antibody as the positive control and labeled drugs as the bridge components. The assay included acid pre-treatment of serum samples to dissociate any antibody:drug complexes present in the sample and improve detection of ADAs in positive samples. ADA testing was performed as outlined in regulatory guidance documents^1,2^ and involved up to three different evaluations: initial screening assay to identify samples potentially positive for ADAs; confirmatory assay to determine whether positive screening assay responses can be inhibited by the presence of excess drug; and a titer procedure to assess ADA levels in confirmed positive samples. Positive responses were determined using a cut point (response threshold) established for patients with atopic dermatitis (AD). Titer values of ADA-positive samples were defined as the maximum final dilution from neat serum that has an assay signal still above the assay cut point (**Table II**). Assay sensitivity in neat serum is 9.9 ng/mL of monoclonal-antibody-positive control. The drug tolerance limit (DTL) in neat serum is approximately 164 μg/mL of dupilumab.

**Neutralizing antibodies (NAbs).** The presence of NAbs was evaluated using a validated competitive ligand-binding assay in ADA-positive serum samples; ADA-negative samples were considered NAb-negative. The assay utilizes biotinylated dupilumab to capture NAbs present in serum samples. The presence of NAbs prevents subsequent binding of biotinylated dupilumab to ruthenium-labeled interleukin-4 receptor alpha (IL-4Rα) in the assay, which results in an assay signal reduction. Samples with percent inhibition greater than the assay cut point are reported as positive for neutralizing activity. Using the assay cut point with a 1% false‑positive rate, assay sensitivity in neat serum is 125 ng/mL of monoclonal-antibody-positive control. DTL in neat serum is approximately 298 ng/mL of dupilumab.

**Dupilumab concentrations in serum.** Serum samples for quantitation of dupilumab were analyzed using a validated enzyme-linked immunosorbent assay, with dupilumab as assay standard and human IL-4Rα as capture reagent.^3^ Concentrations of dupilumab in serum were measured as functional drug, with either one or two available free-binding sites measured (functional drug). The assay does not detect dupilumab when both binding sites are occupied by soluble IL-4Rα or at least one site is bound to membrane-bound IL-4Rα. The lower limit of quantitation of functional dupilumab is 0.0780 mg/L in undiluted human serum.^4^

# Patient narratives of serum sickness or serum sickness-like reactions

**1. Serum sickness in the adult open-label extension (OLE):** The patient was a 30-year-old female who received placebo in SOLO 1 and in SOLO-CONTINUE. She had the event of serum sickness on day 18 after receiving 2 doses of dupilumab 300 mg qw in the OLE. The patient presented to the emergency room with joint pain, fever, tachycardia, headache, and rash. She reported hives and noted that the joint pain was worse on the right side, and reported feeling weak and unable to walk. Trace edema was noted in the lower extremities, and tenderness and swelling of the joints of the fingers, hands, and wrists were noted. A macular rash was noted on the extremities and an ECG showed sinus tachycardia. The patient was treated with vancomycin and cefepime. Her white blood cell count, erythrocyte sedimentation rate, total bilirubin, and C-reactive protein (CRP) were elevated, while her albumin, aspartate aminotransferase (AST), and total creatine kinase were decreased. Urinalysis showed cloudy dark yellow urine with the presence of increased red blood cells (15–19 per high powered field), white blood cells (10–14 per high-powered field), protein (100 mg/dL), bilirubin, ketones (15 mg/dL), moderate blood, many epithelial cells, many bacteria, and hyaline casts. Corrective treatment included vancomycin, cefepime, tramadol hydrochloride, naproxen, omeprazole, prednisone taper, trazodone, calcium pantothenate/nicotinamide/pyridoxine hydrocholoride/riboflavin/thiamine HCl (vitamin B complex), and potassium. A consulting physician noted that the patient had a serum-sickness-like reaction, and it was suspected this was due to study drug. The event resolved on day 21 and discharge medications included naproxen, omeprazole, prednisone taper, tramadol hydrochloride, and trazodone. Study drug was permanently discontinued due to the event, and the event was considered related to the study drug. The patient consistently exhibited a pre-existing persistent low-titer ADA response throughout SOLO 1 and SOLO-CONTINUE (titer range 120–480). The ADA titer from this patient increased from 120 on day 0 (e.g. upon entry into the OLE) to 15,360 at the end of treatment (early termination visit, at approximately 79 days), and then decreased to 7,680 at the end of study visit at day 129. The patient also tested NAb-positive throughout the OLE.

**2. Serum-sickness-like reaction in LIBERTY AD EVALUATE (trial not included in the analysis; NCT02210780):** In this phase 2 randomized placebo-controlled trial, a 38-year-old female with high-titer ADAs (titer of 122,880) in the dupilumab 300 mg weekly (qw) group developed a treatment-emergent serious adverse event (TE-SAE) of serum-sickness-like reaction at day 15, following 3 doses of dupilumab (loading dose dupilumab 600 mg, followed by dupilumab 300 mg qw for 2 weeks), that led to permanent discontinuation of the study drug.^5,6^ The event was recovered/resolved at 21 days after onset. This TE-SAE was considered by the investigator to be moderate in severity and related to the study drug. The patient was ADA-negative at baseline, ADA-positive at study day 15 (titer of 240), and ADA-positive at the early termination visit (study day 22), with a peak titer of 122,880 at the early termination visit. NAbs were not evaluated in this patient.

**3. Serum sickness in LIBERTY AD CAFÉ** **(trial not included in the analysis; NCT02755649):** A 56-year-old White female in the 300 mg qw + topical corticosteroid (TCS) group, with a medical history significant for allergic rhinitis and glaucoma, received a loading dose of 600 mg dupilumab and then experienced diarrhea, headache, and dizziness of mild severity later that day. Symptoms resolved 2 days later. The patient also developed an injection-site reaction (ISR) of moderate severity. The investigator made a diagnosis of serum sickness, and the study drug was temporarily discontinued. Study drug was permanently withdrawn 19 days later, due to the ISR. The patient never received a second dose of the study drug and withdrew from the trial. ADA status was negative at baseline; at the last visit, ADA status was positive with a low titer of 30 and was negative for NAbs.

# TABLE E1. Blood sample timepoints for ADAs and PK assessment

| **Trial** | **ADA sample timepoints (week)** | **PK sample timepoints (week)** |
| --- | --- | --- |
| SOLO 1 and 2 | 0, 2, 4, 8, 16, 28^a^ | 0, 2, 4, 8, 12, 16, 28^a^ |
| CHRONOS | 0, 2, 4, 8, 16, 36, 52, 64^a^ | 0, 2, 4, 8, 12, 16, 20, 28, 36, 44, 52, 60^a^, 64^a^ |
| SOLO-CONTINUE | 0, 4, 12, 24, 36, 48^a^ | 0, 4, 12, 24, 36, 48^a^ |
| OLE | 0, 48, 100, 124, 148, 172, 220, 260 | 0, 48, 100, 124, 260 |
| ADOL | 0, 4, 16, 28^a^ | 0, 2, 4, 8, 12, 16, 28^a^ |
| PEDS | 0, 4, 16, 28^a^ | 0, 4, 8, 12, 16, 24^a^, 28^a^ |
| PRESCHOOL | 0, 16, 28^a^ | 0, 4, 8, 12, 16, 20^a^, 28^a^ |
| PED-OLE | 0, 16, 52, 76, 104, 152, 200, 260 | 0, 16, 52, 76, 104, 152, 200, 260 |

*ADA*, anti-drug antibody; *PK*, pharmacokinetics/drug concentration.

Week 0 = baseline. Blood samples for PK and ADA assessment were collected upon early termination in all studies. ADAs in PEDS were not evaluated at week 4 unless results were positive at week 16.

^a^Post-treatment follow-up period; only applicable for patients not enrolling directly into the next study.

# TABLE E2. Patient ADA/NAb status and maximum titer category for long-term trials: SOLO-CONTINUE, OLE, and PED-OLE; ADA analysis set

|  | **Adult trials** | | | | **PED-OLE^a.b^** | |
| --- | --- | --- | --- | --- | --- | --- |
|  | **SOLO-CONTINUE^b^** | | | **OLE^b^** | **Parent study:**  **ADOL** | **Parent study:**  **PEDS** |
| **ADA category, n (%)** | **Placebo^c^**  **(n = 80)** | **Dupilumab**  **300 mg q2w**  **(n = 70)** | **Dupilumab 300 mg qw**  **(n = 83)** | **Dupilumab**  **300 mg qw**  **(n = 1,751)** | **Dupilumab 300 mg q4w^d^**  **(n = 196)** | **Dupilumab 300 mg q4w^d^**  **(n = 327)** |
| PRE | 3 (3.8) | 4 (5.7) | 1 (1.2) | 85 (4.9) | 7 (3.6) | 8 (2.4) |
| TB response | 1 (1.3) | 0 | 0 | 7 (0.4) | 0 | 0 |
| TE response | 9 (11.3) | 2 (2.9) | 0 | 100 (5.7) | 24 (12.2) | 9 (2.8) |
| Persistent | 0 | 0 | 0 | 15 (0.9) | 3 (1.5) | 2 (0.6) |
| Transient | 0 | 0 | 0 | 76 (4.3) | 5 (2.6) | 3 (0.9) |
| Indeterminate | 9 (11.3) | 2 (2.9) | 0 | 9 (0.5) | 16 (8.2) | 4 (1.2) |
| ADA-negative^e^ | 67 (83.8) | 64 (91.4) | 82 (98.8) | 1,559 (89.0) | 165 (84.2) | 310 (94.8) |
| Maximum ADA titer^f^ |  |  |  |  |  |  |
| Low (<1,000) | 9 (11.3) | 2 (2.9) | 0 | 93 (5.3) | 21 (10.7) | 8 (2.4) |
| Moderate (≥1,000 and ≤10,000) | 1 (1.3) | 0 | 0 | 11 (0.6) | 2 (1.0) | 0 |
| High (>10,000) | 0 | 0 | 0 | 3 (0.2) | 1 (0.5) | 1 (0.3) |
| NAb analysis set | 79 (98.8) | 69 (98.6) | 83 (100) | 1,751 (100) | 189 (96.4) | 322 (98.5) |
| NAb-negative | 75 (93.8) | 69 (98.6) | 82 (98.8) | 1,705 (97.4) | 179 (91.3) | 322 (98.5) |
| NAb-positive | 4 (5.0) | 0 | 1 (1.2) | 46 (2.6) | 10 (5.1) | 0 |

*ADA*, anti-dupilumab antibody; *n*, ADA analysis set; *NAb*, neutralizing antibody; *PRE*, pre-existing immunogenicity detected at parent study baseline; *qw*, once weekly; *q2w*, every 2 weeks; *q4w*, every 4 weeks; *TB*, treatment-boosted; *TE*, treatment-emergent.

^a^Subset of patients who were previously enrolled in ADOL or PEDS.

^b^ADAs that were observed during PED-OLE or OLE were defined as TE if the patient was ADA-negative at baseline of parent study and had TE-ADA in parent study, or if new TE-ADA in OLE. Baseline status in SOLO-CONTINUE, OLE, or PED-OLE is considered as baseline in parent study for determination of ADA status.

^c^Patients in SOLO-CONTINUE received dupilumab in a prior study; thus, these patients were not dupilumab-naïve.

^d^Some patients were uptitrated to 200/300 mg q2w during the course of the study.

^e^ADA-negative: all samples were negative in the ADA assay.

^f^Maximum titer category is reported only for patients who were ADA-positive at any time. The titer value of an ADA-positive sample is defined as the maximum dilution of the sample that has an assay signal still above the assay cut point.

# TABLE E3. Safety outcomes for long-term trials: SOLO-CONTINUE, OLE, and PED-OLE

|  |  | **Adult trials** | | | | **PED-OLE^a,b^** | |
| --- | --- | --- | --- | --- | --- | --- | --- |
|  |  | **SOLO-CONTINUE^b^** | | | **OLE^b^** | **Parent study:**  **ADOL** | **Parent study:**  **PEDS** |
| **Number of patients with at least one such event, n1/N (%)** | **ADA status^c^** | **Placebo**  **(n = 82)^d^** | **Dupilumab**  **300 mg q2w**  **(n = 80)** | **Dupilumab 300 mg qw**  **(n = 87)** | **Dupilumab**  **300 mg qw**  **(n = 1,751)** | **Dupilumab 300 mg q4w^e^**  **(n = 201)** | **Dupilumab 300 mg q4w^e^**  **(n = 335)** |
| **Overview** | | | | | | | |
| Any TEAE | ADA+ | 10/10 (100) | 1/2 (50.0) | 0 | 84/107 (78.5) | 13/24 (54.2) | 8/9 (88.9) |
|  | ADA− | 56/70 (80.0) | 54/69 (78.3) | 59/83 (71.1) | 1362/1,644 (82.8) | 74/172 (43.0) | 179/318 (56.3) |
|  | Missing | 1/2 (50.0) | 3/9 (33.3) | 1/4 (25.0) | n/a | 0/5 | 0/8 |
| TEAE related to study drug | ADA+ | 1/10 (10.0) | 1/2 (50.0) | 0 | 35/107 (32.7) | 1/24 (4.2) | 1/9 (11.1) |
|  | ADA− | 21/70 (30.0) | 17/69 (24.6) | 13/83 (15.7) | 518/1,644 (31.5) | 11/172 (6.4) | 43/318 (13.5) |
|  | Missing | 0 | 1/9 (11.1) | 0 | 0 | 0 | 0 |
| TEAE causing permanent discontinuation of study drug | ADA+ | 0 | 0 | 0 | 7/107 (6.5) | 0 | 0 |
|  | ADA− | 2/70 (2.9) | 0 | 0 | 50/1,644 (3.0) | 0 | 2/318 (0.6) |
|  | Missing | 1/2 (50.0) | 0 | 0 | n/a | 0 | 0 |
| Death | ADA+ | 0 | 0 | 0 | 0 | 0 | 0 |
|  | ADA− | 0 | 0 | 0 | 2/1,644 (0.1) | 0 | 0 |
|  | Missing | 0 | 0 | 0 | n/a | 0 | 0 |
| Any TE-SAE | ADA+ | 0 | 0 | 0 | 7/107 (6.5) | 1/24 (42) | 0 |
|  | ADA− | 1/70 (1.4) | 4/69 (5.8) | 2/83 (2.4) | 143/1,644 (8.7) | 0 | 4/318 (1.3) |
|  | Missing | 0 | 0 | 0 | n/a | 0 | 0 |
| TE-SAE related to study drug | ADA+ | 0 | 0 | 0 | 4/107 (3.7) | 0 | 0 |
|  | ADA− | 0 | 0 | 0 | 16/1,644 (1.0) | 0 | 0 |
|  | Missing | 0 | 0 | 0 | n/a | 0 | 0 |
| TE-SAE causing permanent discontinuation of study drug | ADA+ | 0 | 0 | 0 | 2/107 (1.9) | 0 | 0 |
|  | ADA− | 0 | 0 | 0 | 22/1,644 (1.3) | 0 | 0 |
|  | Missing | 0 | 0 | 0 | n/a | 0 | 0 |
| **Adverse events of special interest** | | | | | | | |
| Serum sickness/serum-sickness-like reactions^g^ | ADA+ | 0 | 0 | 0 | 1/107 (0.9) | 0 | 0 |
|  | ADA− | 0 | 0 | 0 | 0 | 0 | 0 |
|  | Missing | 0 | 0 | 0 | n/a | 0 | 0 |
| Anaphylaxis / anaphylactic reaction MedDRA SMQ narrow ^h^ | ADA+ | 0 | 0 | 0 | 0 | 0 | 0 |
|  | ADA− | 0 | 0 | 1/83 (1.2) | 4/1,644 (0.2) | 0 | 3/318 (0.9) |
|  | Missing | 0 | 0 | 0 | n/a | 0 | 0 |
| ISRs^f^ lasting >24 hours^i^ | ADA+ | 0 | 0 | 0 | 0 | n/a | n/a |
|  | ADA− | 0 | 0 | 0 | 0 | n/a | n/a |
|  | Missing | 0 | 0 | 0 | 0 | n/a | n/a |

*ADA*, anti-drug antibody; *ADA−,* ADA-negative; *ADA+,* ADA-positive; *HLT*, MedDRA High Level term; *ISR*, injection-site reaction; *MedDRA*, Medical Dictionary for Regulatory Activities; *N*, number of patients in the ADA group for each dose group; *n*, number of patients in dose group, based on the safety analysis set; *n1*, number of patients with an event; *n/a*, not available; *PRE*, pre-existing immunoreactivity; *PT*, MedDRA Preferred Term; qw, once weekly; *q2w*, every 2 weeks; *q4w*, every 4 weeks; *SMQ*, standardized MedDRA query; *TB*, treatment-boosted; *TE*, treatment-emergent; *TEAE*, treatment-emergent adverse event; *TE-SAE*, treatment-emergent serious adverse event.

^a^Subset of patients who were previously enrolled in ADOL or PEDS.

^b^For SOLO-CONTINUE, OLE, and PED-OLE, baseline in the parent studies was considered baseline for determination of ADA status.

^c^For the purpose of the safety analysis, ADA+ included TE or TB, and ADA− included either true negative or PRE.

^d^Placebo-treated patients in SOLO-CONTINUE had previously received dupilumab in the parent study.

^e^Some patients were uptitrated to 200/300 mg q2w during the course of the study.

^f^MedDRA HLT.

^g^MedDRA PTs.

^h^MedDRA PTs included in in the MedDRA “anaphylaxis/anaphylactic reaction SMQ narrow”.

^i^ISR s lasting >24 hours were not defined in PED-OLE. In the adult OLE, these data were no longer collected following amendment 7 of the study protocol (amendment instituted June 2, 2017).

# TABLE E4. Safety outcomes (TE-SAEs and permanent discontinuation of study drug) by ADA titer (high, moderate, low). A. RCTs; B. Long-term trials

**A. RCTs**

|  |  | **Adult trials** | | | | | | **Pediatric trials** | | | | | | | |
| --- | --- | --- | --- | --- | --- | --- | --- | --- | --- | --- | --- | --- | --- | --- | --- |
| **Number of ADA-positive patients with ≥1 such event, n1/N (%)** | **Trial(s) (treatment period)** | **SOLO 1 & 2 pooled**  **(16 weeks)** | | | **CHRONOS**  **(52 weeks)** | | | **ADOL (12–17 years)**  **(16 weeks)** | | **PEDS (6–11 years)**  **(16 weeks)** | | | | **PRESCHOOL (6 months–5 years) (16 weeks)** | |
| **Type of event** | **ADA titer^a^** | **PBO**  **(n = 456)** | **DPL**  **300 mg q2w**  **(n = 465)** | **DPL**  **300 mg qw**  **(n = 455)** | **PBO + TCS**  **(n = 315)** | **DPL**  **300 mg q2w + TCS**  **(n = 110)** | **DPL**  **300 mg qw + TCS**  **(n = 315)** | **PBO**  **(n = 85)** | **DPL 200/300 mg q2w**  **(n = 82)** | **PBO + TCS**  **(n = 120)** | **DPL**  **200 mg q2w + TCS (≥30 kg)**  **(n = 59)** | **DPL**  **300 mg q4w + TCS (<30 kg)**  **(n = 60)** | **DPL**  **300 mg q4w + TCS (any weight)^b^**  **(n = 120)** | **PBO + TCS**  **(n = 78)** | **DPL**  **200/300 mg q4w + TCS**  **(n = 83)** |
| TE-SAE | High | n/a | n/a | 0/2 (0) | n/a | n/a | n/a | n/a | n/a | n/a | n/a | n/a | n/a | n/a | n/a |
|  | Moderate | n/a | 0/3 (0) | 0/3 (0) | n/a | 0/1 (0) | 0/2 (0) | n/a | 0/2 (0) | n/a | n/a | n/a | n/a | n/a | n/a |
|  | Low | 0/8 (0) | 0/30 (0) | 0/7 (0) | 0/24 (0) | 0/9 (0) | 2/17 (11.8)^b^ | 0/3 (0) | 0/11 (0) | 0/2 (0) | 0/3 (0) | n/a | n/a | n/a | 0/1 (0) |
| Permanent discontinuation of study drug | High | n/a | n/a | 0/2 (0) | n/a | n/a | n/a | n/a | n/a | n/a | n/a | n/a | n/a | n/a | n/a |
|  | Moderate | n/a | 0/3 (0) | 0/3 (0) | n/a | 0/1 (0) | 0/2 (0) | n/a | 0/2 (0) | n/a | n/a | n/a | n/a | n/a | n/a |
|  | Low | 1/8 (12.5) | 0/30 (0) | 1/7 (14.3) | 1/24 (4.2)^c^ | 0/9 (0) | 3/17 (17.6)^d^ | 0/3 (0) | 0/11 (0) | 0/2 | 0/3 (0) | n/a | n/a | n/a | 0/1 (0) |

*ADA*, anti-drug antibody; *DPL*, dupilumab; *MedDRA*, Medical Dictionary for Regulatory Activities; *N*, number of patients in titer category; *n1*, number of patients with ≥1 event; *n/a*, no patients were in the titer category; *PBO*, placebo; *PT*, MedDRA Preferred Term; *qw*, once weekly; *q2w*, every 2 weeks; *q4w*, every 4 weeks; *RCT*, randomized controlled trial; *TCS*, topical corticosteroid(s); *TE-SAE*, treatment-emergent adverse event.

All events listed in footnotes are MedDRA PTs.

^a^Titers were defined as low (<1,000), moderate (≥1,000 to <10,000), and high (≥10,000).

^b^One patient reported cystoid macular edema, and one reported rash maculo-papular.

^c^One patient reported dermatitis atopic, fatigue, malaise, and pain.

^d^One patient each reported allergic keratitis, cystoid macular edema, and rash maculo-papular.

**B. Long-term trials: SOLO-CONTINUE, OLE, and PED-OLE.**

| **Number of ADA-positive patients with at least one such event, n1/N (%)** |  | **Adult trials** | | | | **Pediatric trial** | |
| --- | --- | --- | --- | --- | --- | --- | --- |
|  | **Trial(s) (treatment period)** | **SOLO-CONTINUE^b^**  **(36 weeks)** | | | **OLE^b^**  **(up to 3 years)** | **PED-OLE^a,b^**  **(up to 1 year)** | **PED-OLE^a,b^**  **(up to 1 year)** |
| **Type of event** | **ADA titer^c^** | **Placebo**  **(n = 82)^d^** | **Dupilumab**  **300 mg q2w**  **(n = 80)** | **Dupilumab 300 mg qw**  **(n = 87)** | **Dupilumab**  **300 mg qw**  **(n = 1,751)** | **Dupilumab 300 mg q4w^e^**  **(n = 201)** | **Dupilumab 300 mg q4w^e^**  **(n = 335)** |
| TE-SAE | High | n/a | n/a | n/a | 1/3 (33.3)^f^ | 1/1 (100)^g^ | 0/1 (0) |
|  | Moderate | 0/1 (0) | n/a | n/a | 0/11 (0) | 0/2 (0) | n/a |
|  | Low | 0/9 (0) | 0/2 (0) | n/a | 6/93 (6.5)^h^ | 0/21 (0) | 0/8 (0) |
| Permanent discontinuation of study drug | High | n/a | n/a | n/a | 2/3 (66.7)^i^ | n/a | 0/1 (0) |
|  | Moderate | 0/1 (0) | n/a | n/a | 0/11 (0) | n/a | n/a |
|  | Low | 0/9 (0) | 0/2 (0) | n/a | 5/93 (5.4)^j^ | n/a | 0/8 (0) |

*ADA*, anti-drug antibody; *N*, number of patients in titer category; *n1*, number of patients with ≥1 event; *n/a*, no patients were in the titer category; *qw*, weekly; *q2w*, every 2 weeks; *q4w*, every 4 weeks; *TE-SAE*, treatment-emergent adverse event.

^a^Subset of patients who were previously enrolled in ADOL or PEDS.

^b^For SOLO-CONTINUE, OLE, and PED-OLE, baseline in the parent studies was considered baseline for determination of ADA status.

^c^Titers were defined as low (<1,000), moderate (≥1,000 to <10,000), and high (≥10,000).

^d^Placebo-treated patients in SOLO-CONTINUE had previously received dupilumab in the parent study.

^e^Some patients were uptitrated to 200/300 mg q2w during the course of the study.

^f^One patient reported serum sickness.

^g^One patient reported injection-site cellulitis.

^h^One patient each reported bronchitis, cellulitis, lung infection, breast cancer, small cell lung cancer, optic neuropathy, device dislocation, and migraine. Some patients may have reported more than 1 event.

^i^One patient each reported serum sickness and dermatitis psoriasiform.

^j^Two patients reported conjunctivitis, 1 reported sarcoidosis, 1 reported small cell lung cancer, and 1 reported psoriasis.

# **FIG E1.** Proportion of patients pooled from SOLOs, CHRONOS, ADOL, PEDS, and PRESCHOOL with TE or TB ADAs that were NAb-positive, by maximum titer category.

#
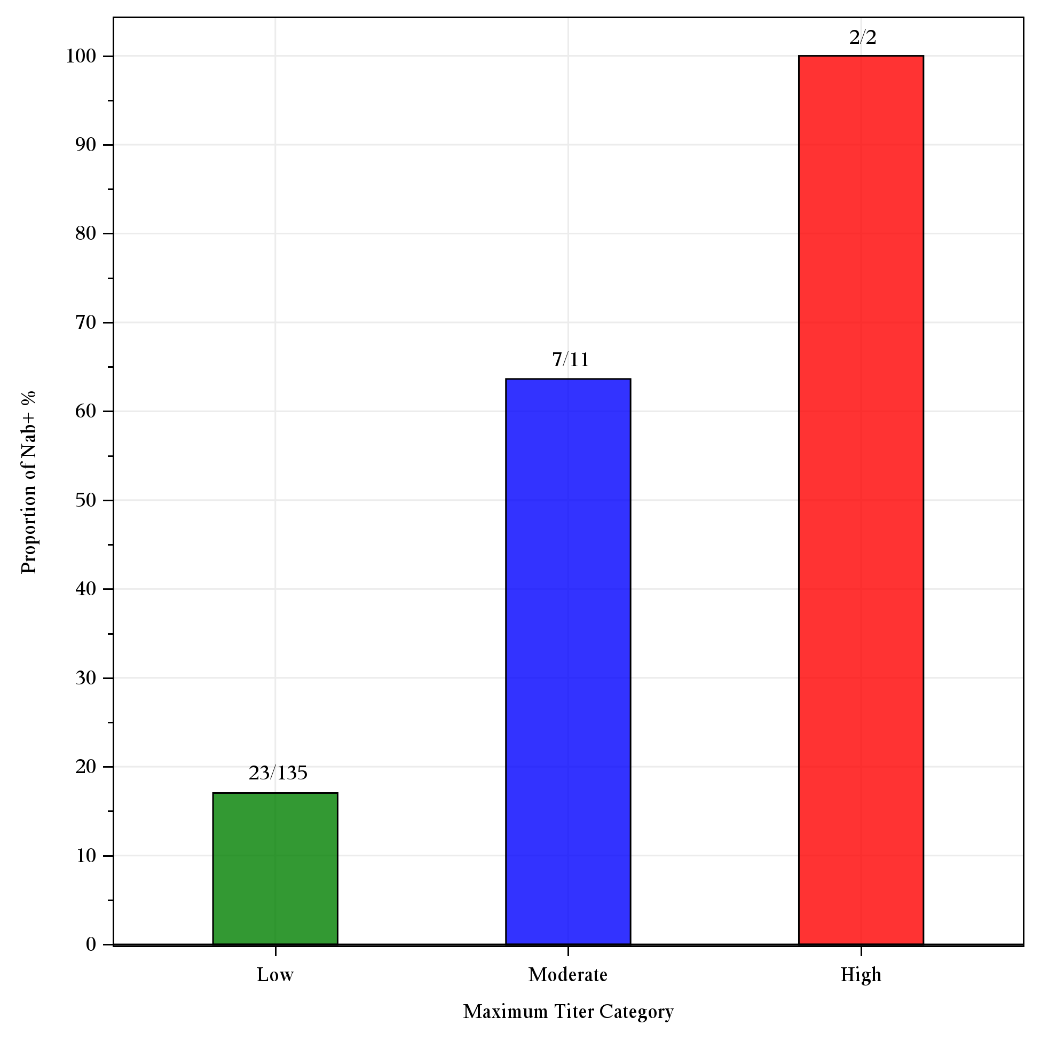


# ADA, anti-dupilumab antibody; n, number of NAb-positive patients in titer category; N, number of patients in titer category; NAb, neutralizing antibody; TB, treatment-boosted; TE, treatment-emergent.

# **FIG E2. Individual concentrations of functional dupilumab in serum by nominal time and maximum ADA titer category**.^a^


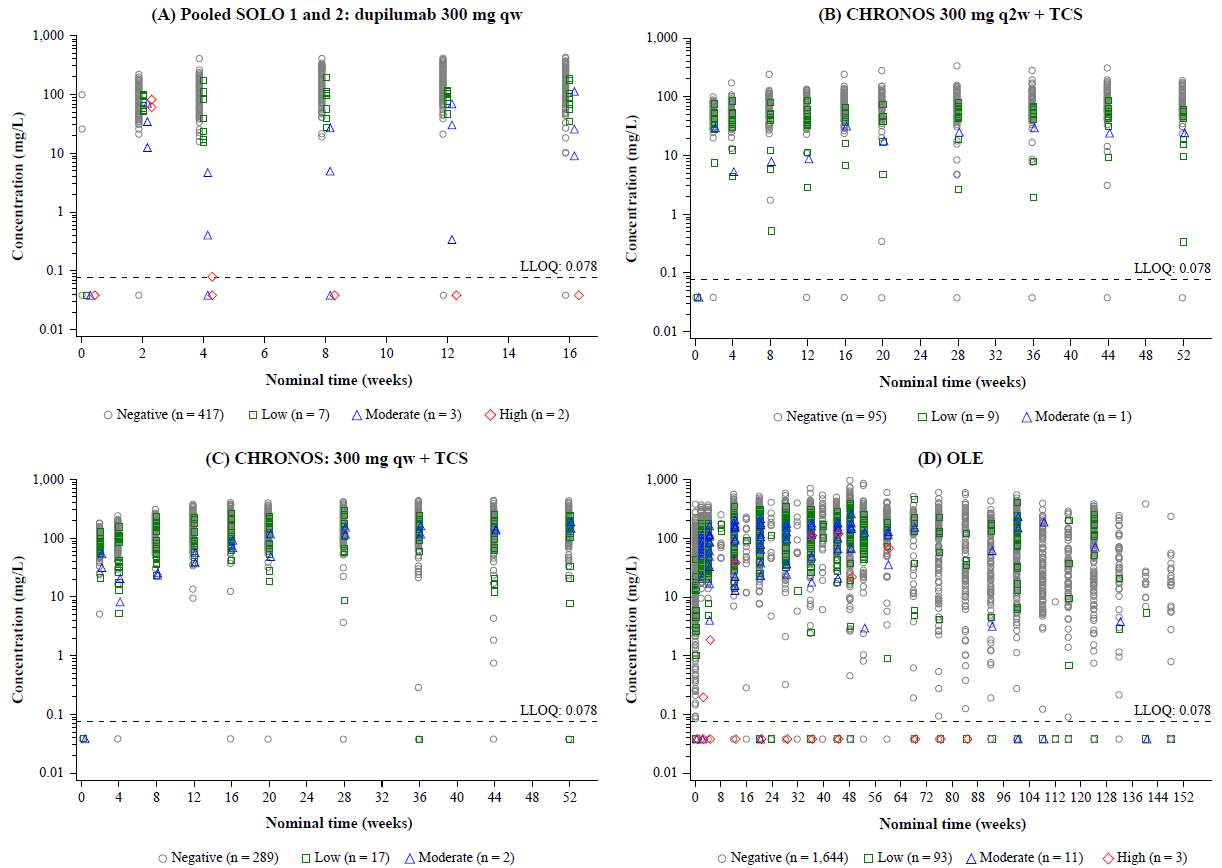


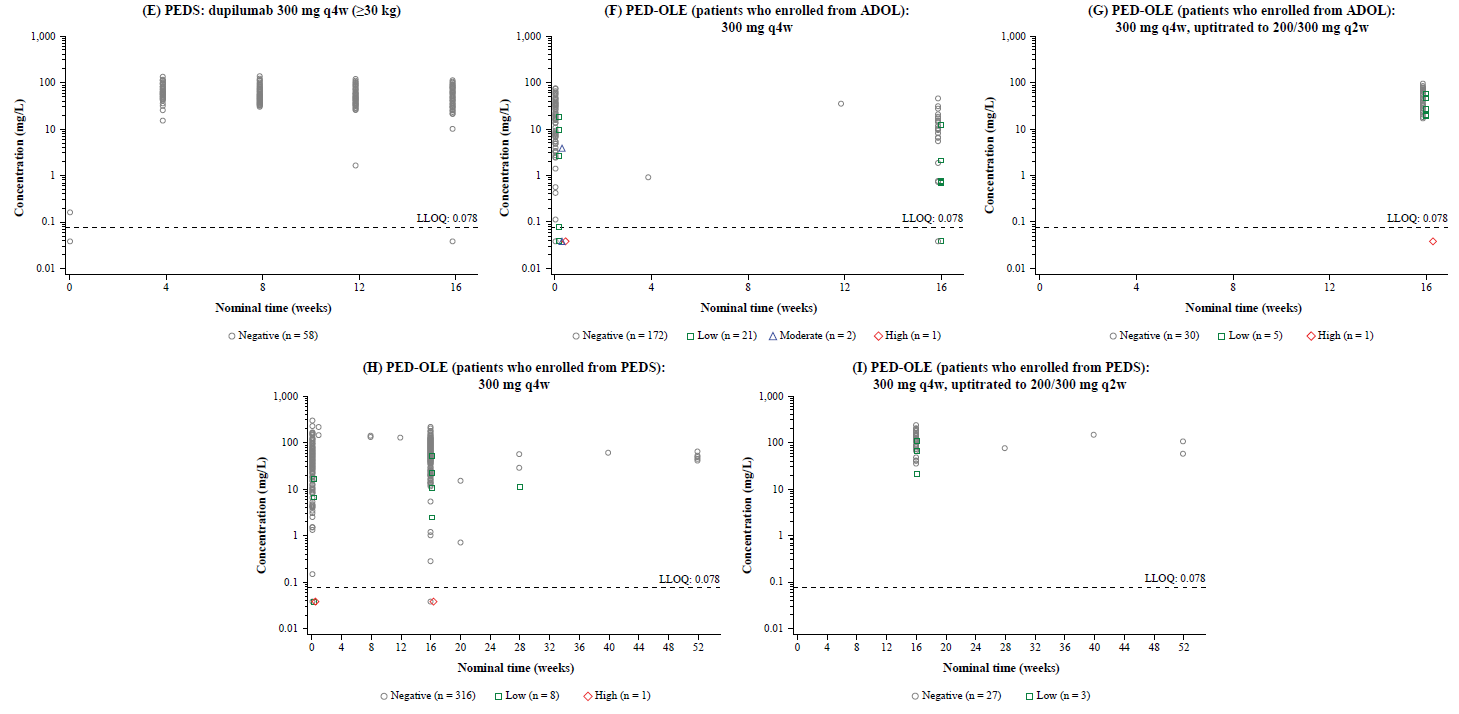


**(A)** SOLO 1 and 2 pooled, dupilumab 300 mg qw. **(B)** CHRONOS 300 mg q2w + TCS. **(C)** CHRONOS: 300 qw + TCS. **(D)** OLE, 300 mg qw. **(E)** PEDS: dupilumab 300 mg q4w (≥30 kg). **(F)** PED-OLE (patients who enrolled from ADOL): 300 mg q4w. **(G)** PED-OLE (patients who enrolled from ADOL): 300 mg q4w, uptitrated to 200/300 mg q2w. **(H)** PED-OLE (patients who enrolled from PEDS): 300 mg q4w. **(I)** PED-OLE (patients who enrolled from PEDS): 300 mg q4w, uptitrated to 200/300 mg q2w.

Concentrations below the LLOQ (horizontal dashed line) were set to LLOQ/2. Concentration results are jittered by maximum-titer category on the X-axis for better data presentation.

*ADA*, anti-drug antibody; *LLOQ*, lower limit of quantitation; *qw*, once weekly; *q2w*, every 2 weeks; *q4w*, every 4 weeks; *TCS*, topical corticosteroid(s).

^a^Low = ADA titer <1,000; moderate = ADA titer ≥1,000 to ≤10,000; high = ADA titer >10,000.

# FIG E3. Individual log-scaled concentration of functional dupilumab in serum and EASI percent change from baseline over time by ADA titer in patients with high- or moderate-titer ADAs in the adult OLE


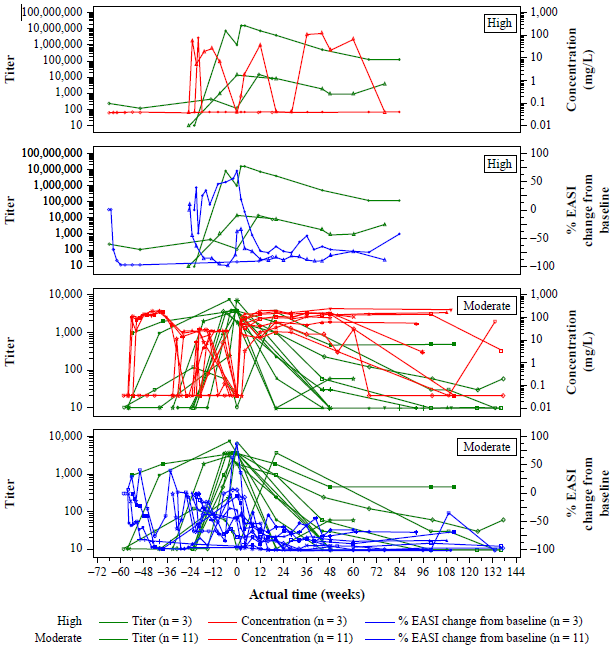


Baseline records from parent studies were used to derive maximum titer category and ADA status. The titer was imputed as 10 for negative ADA results for data presentation purposes.

*ADA*, anti-drug antibody; *EASI*, Eczema Area and Severity Index; *n*, number of participants.

# **FIG E4. Efficacy: percent change in EASI from baseline by nominal time and maximum ADA titer category.**


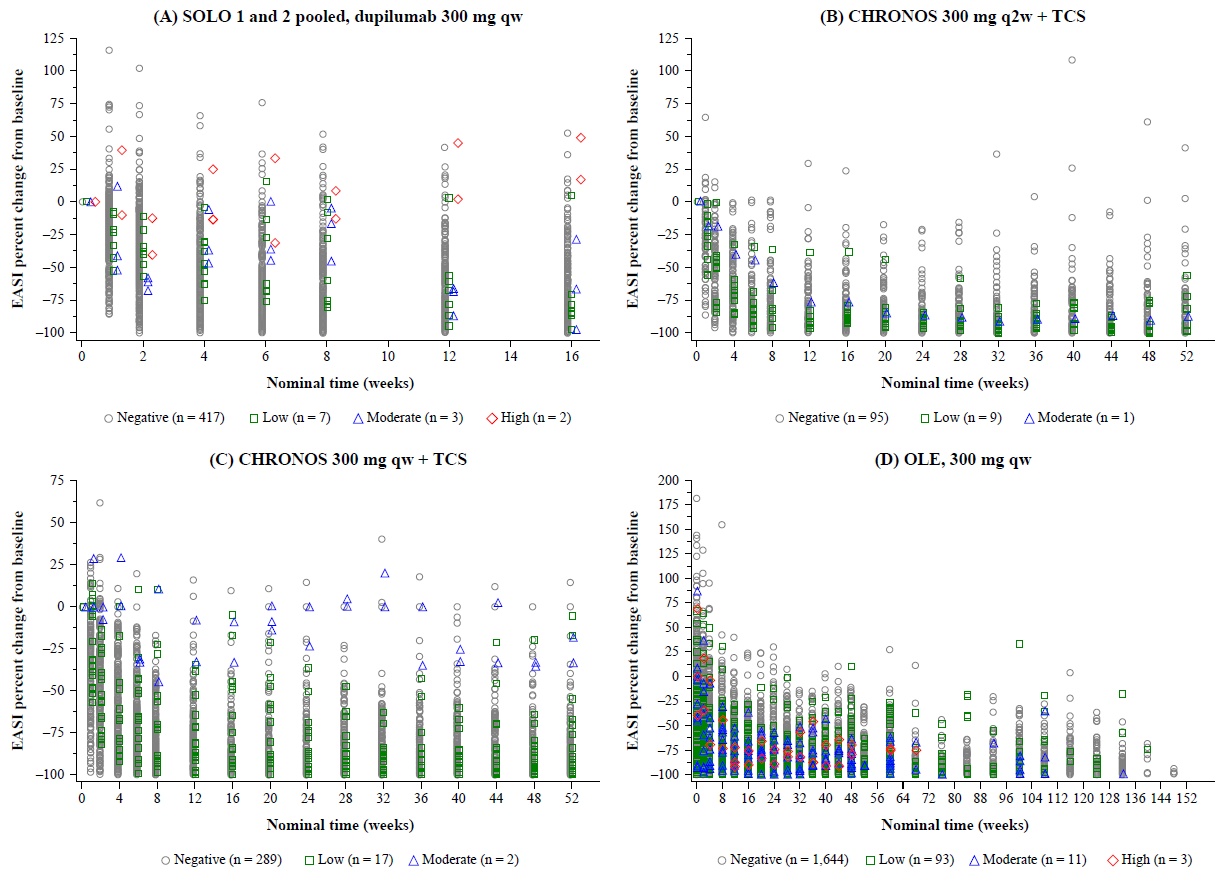


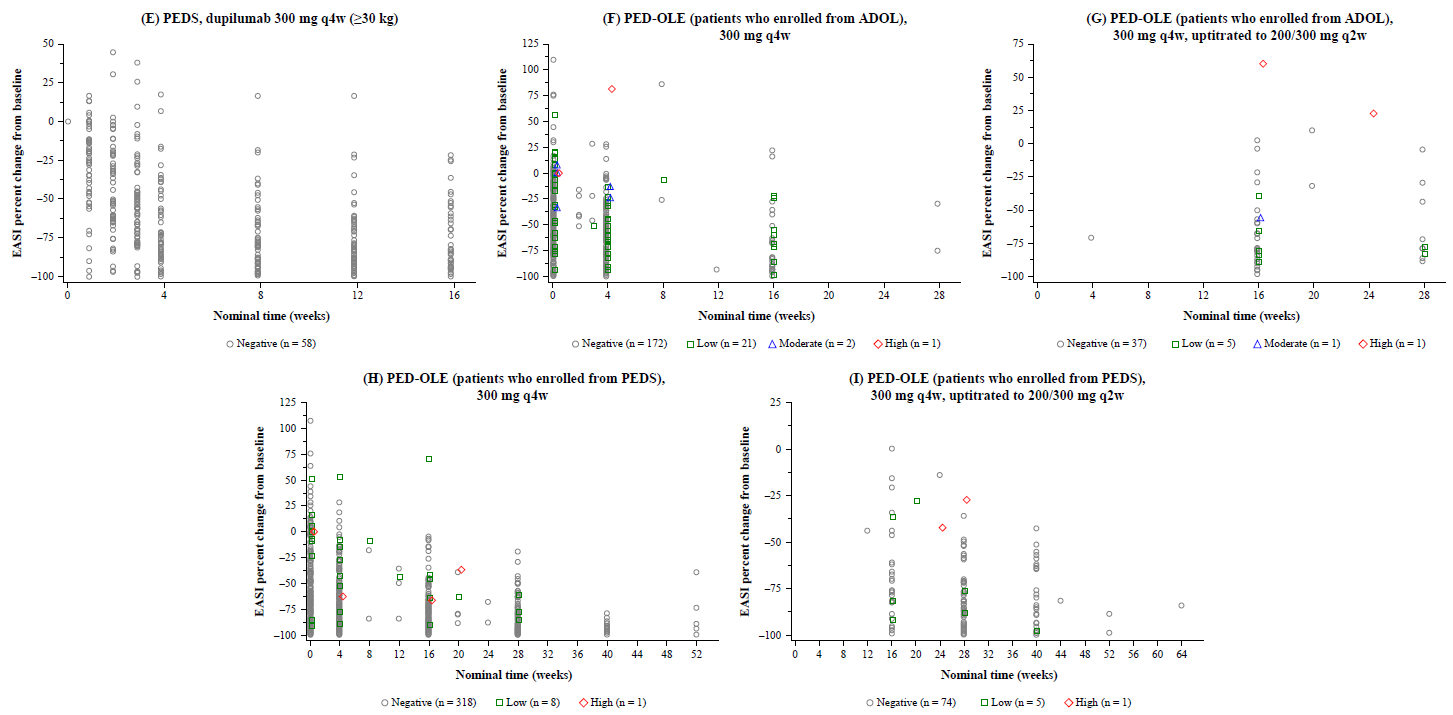


**(A)** SOLO 1 and 2 pooled, dupilumab 300 mg qw. **(B)** CHRONOS 300 mg q2w + TCS. **(C)** CHRONOS 300 qw + TCS. **(D)** OLE, 300 mg qw. **(E)** PEDS, dupilumab 300 mg q4w (≥30 kg). **(F)** PED-OLE (patients who enrolled from ADOL), 300 mg q4w. **(G)** PED-OLE (patients who enrolled from ADOL), 300 mg q4w, uptitrated to 200/300 mg q2w. **(H)** PED-OLE (patients who enrolled from PEDS), 300 mg q4w. **(I)** PED-OLE (patients who enrolled from PEDS), 300 mg q4w, uptitrated to 200/300 mg q2w.

Results are jittered by ADA maximum titer category on the X-axis for better data presentation.

*ADA*, anti-dupilumab antibody; *EASI*, Eczema Area and Severity Index; *qw*, weekly; *q2w*, every 2 weeks; *q4w*, every 4 weeks; *TCS*, topical corticosteroid(s).

^a^Low = ADA titer <1,000; moderate = ADA titer ≥1,000 to ≤10,000; high = ADA titer >10,000.

# Supplementary references

1. US Food and Drug Administration (FDA). Immunogenicity Testing of Therapeutic Protein Products—Developing and Validating Assays for Anti-Drug Antibody Detection. 2019. Available at: <https://www.fda.gov/regulatory-information/search-fda-guidance-documents/immunogenicity-testing-therapeutic-protein-products-developing-and-validating-assays-anti-drug>. Accessed October 9, 2023.
2. European Medicines Agency (EMA). Guideline on Immunogenicity Assessment of Therapeutic Proteins. 2017. Available at: <https://www.ema.europa.eu/en/documents/scientific-guideline/guideline-immunogenicity-assessment-therapeutic-proteins-revision-1_en.pdf>. Accessed July 7, 2022.
3. Kamal MA, Davis JD, Kovalenko P, et al. Pharmacokinetics, pharmacodynamics, and exposure–efficacy of dupilumab in adults with atopic dermatitis. Clin Transl Sci 2022;15:2342-2354. doi: 10.1111/cts.13363.
4. Davis JD, Bansal A, Hassman D, Akinlade B, Li M, Li Z, et al. Evaluation of potential disease-mediated drug-drug interaction in patients with moderate-to-severe atopic dermatitis receiving dupilumab. Clin Pharmacol Ther 2018;104:1146-54. doi: 10.1002/cpt.1058.
5. US Food and Drug Administration (FDA). DUPIXENT® (dupilumab). Prescribing Information. 2022. Available at: https://www.regeneron.com/downloads/dupixent_fpi.pdf. Accessed September 14, 2023.
6. Blauvelt A, Simpson E, Tyring S, Purcell LA, Shumel B, Petro CD, et al. Dupilumab does not affect correlates of vaccine-induced immunity: a randomized, placebo-controlled trial in adults with moderate-to-severe atopic dermatitis. J Am Acad Dermatol 2019;80:158-67.e1. doi: 10.1016/j.jaad.2018.07.048.
7. Thaçi D, Simpson EL, Beck LA, Bieber T, Blauvelt A, Papp K, et al. Efficacy and safety of dupilumab in adults with moderate-to-severe atopic dermatitis inadequately controlled by topical treatments: a randomised, placebo-controlled, dose-ranging phase 2b trial. Lancet 2016;387:40-52.
